# Identifying and understanding benefits associated with return-on-investment from large-scale healthcare Quality Improvement programmes: an integrative systematic literature review

**DOI:** 10.1186/s12913-022-08171-3

**Published:** 2022-08-24

**Authors:** S’thembile Thusini, Maria Milenova, Noushig Nahabedian, Barbara Grey, Tayana Soukup, Claire Henderson

**Affiliations:** 1 King’s College London, London, UK; 2 South London and Maudsley NHS Foundation Trust, London, UK

**Keywords:** Quality Improvement, QI programmes, Costs and benefits, Return on Investment, QI business case

## Abstract

**Background:**

We previously developed a Quality Improvement (QI) Return-on-Investment (ROI) conceptual framework for large-scale healthcare QI programmes. We defined ROI as any monetary or non-monetary value or benefit derived from QI. We called the framework the QI-ROI conceptual framework. The current study describes the different categories of benefits covered by this framework and explores the relationships between these benefits.

**Methods:**

We searched Medline, Embase, Global health, PsycInfo, EconLit, NHS EED, Web of Science, Google Scholar, organisational journals, and citations, using ROI or returns-on-investment concepts (e.g., cost–benefit, cost-effectiveness, value) combined with healthcare and QI. Our analysis was informed by Complexity Theory in view of the complexity of large QI programmes. We used Framework analysis to analyse the data using a preliminary ROI conceptual framework that was based on organisational obligations towards its stakeholders. Included articles discussed at least three organisational benefits towards these obligations, with at least one financial or patient benefit. We synthesized the different QI benefits discussed.

**Results:**

We retrieved 10 428 articles. One hundred and two (102) articles were selected for full text screening. Of these 34 were excluded and 68 included. Included articles were QI economic, effectiveness, process, and impact evaluations as well as conceptual literature. Based on these literatures, we reviewed and updated our QI-ROI conceptual framework from our first study. Our QI-ROI conceptual framework consists of four categories: 1) organisational performance, 2) organisational development, 3) external outcomes, and 4) unintended outcomes (positive and negative). We found that QI benefits are interlinked, and that ROI in large-scale QI is not merely an end-outcome; there are earlier benefits that matter to organisations that contribute to overall ROI. Organisations also found positive aspects of negative unintended consequences, such as learning from failed QI.

**Discussion and conclusion:**

Our analysis indicated that the QI-ROI conceptual framework is made-up of multi-faceted and interconnected benefits from large-scale QI programmes. One or more of these may be desirable depending on each organisation’s goals and objectives, as well as stage of development. As such, it is possible for organisations to deduce incremental benefits or returns-on-investments throughout a programme lifecycle that are relevant and legitimate.

**Supplementary Information:**

The online version contains supplementary material available at 10.1186/s12913-022-08171-3.

## Introduction

Health services worldwide are faced with challenges to improve the safety and quality of care whilst managing rising healthcare costs [1–4]. One way to improve healthcare quality is through Quality Improvement (QI). QI is a systematic approach to improving healthcare quality as well as strengthening health systems and reducing costs [5, 6]. QI uses sets of methods such as Lean and Plan-Do-Study-Act (PDSA) [7]. These methods often incorporate analysis, improvement or reconfiguring, and monitoring of systems. QI is guided by Implementation and Improvement Sciences in the targeted design of improvement strategies to maximise programmes’ success [8]. QI can be implemented as small projects or large programmes aimed at benefiting entire organisations or health systems [9, 10]. Healthcare is a complex system as it involves connections, actions and interactions of multiple stakeholders and processes [11]. Therefore, QI in healthcare is a complex intervention. This complexity can be costly.

QI may require significant investment to implement and maintain [12, 13]. QI implementers must therefore demonstrate its value to help leaders justify and account for their investment decisions [14, 15]. QI outcomes are assessed through programme evaluations, comparative research, and economic evaluations such as Return on Investment (ROI). ROI is increasingly being recommended for evaluating or forecasting financial returns (making a business case) for healthcare programmes [16, 17]. Originally from accounting and economics, ROI methods calculate a programme’s costs against its benefits [18]. All perceived programme benefits must be converted to money (monetised) and reported as a single ratio or percentage, e.g., ROI of 1:1 means a 100% profit was made [19]. A favourable ROI is where a positive estimation of a financial return from an investment can be made [19, 20]. However, most healthcare benefits are not amenable to monetisation [20]. Additionally, healthcare QI programmes do not often make a profit or save costs [21]. This raises questions of ROI utility in QI programmes.

ROI was introduced into healthcare as a simple objective measure of a programme’s success [16]. However, in practice, ROI methodology has been found to be complicated and sophisticated [22]. ROI has also been found to misrepresent reality due to its inability to incorporate certain crucial programme outcomes that are valued in healthcare [23]. The concerns over ROI have resulted in various attempts to refashion it. Today, there are ROI methods that encourage detailing of non-monetisable qualitative benefits in some way in addition to monetised benefits [24, 25]. However, these methods still prioritise monetisable benefits [19, 20]. As such, some have referred to ROI as insincere and synthetic [24, 26].

The suitability of ROI as a method for evaluating the value of QI in healthcare and other service industries has been contested for decades [23, 27–32]. Within and outside healthcare, others have requested a ‘return to value’ rather a focus on financial outcomes [33] or renamed ROI as ROQ ‘return on quality’ where quality and not profit is favoured [34]. This hints at ROI being a concept. As a concept, ROI encapsulates mental abstractions of how costs and benefits are perceived [35, 36]. Thus, the apparent lack of ROI acceptance in healthcare suggests a need to understand ROI as a concept of a return-on-investment. Understanding the meaning of concepts in research is deemed a crucial step in advancing scientific inquiry [36].

This report is the second part of a larger study on the concept and determinants of ROI from large-scale healthcare QI. The current and previous studies were to develop the ROI concept and a framework for understanding the ROI concept in the healthcare context. The third study will focus on the determinants. In the first part (under submission), we developed the QI-ROI concept by differentiating ROI from similar concepts. In that study, we found that patient outcomes were seen as of primary importance. In addition, several other organisational benefits including financial benefits were also seen as important. We concluded that ROI in healthcare QI represents any valued benefit. We translated this conceptualisation as follows: attaining a return-on-investment whatever that is, is valued and therefore of benefit, and any benefit is of value in and of itself. We then proposed a framework for analysis of return-on-investment from QI programmes. We called this a QI-ROI conceptual framework.

In the current study, we sought to deepen our understanding of the QI-ROI concept. Gelman and Kalish stated that “concepts correspond to categories of things in the real world and are embedded in larger knowledge structures…the building blocks of ideas” [35] (p. 298). Therefore, in the current study, we aimed to search for these building blocks of the QI-ROI concept. The objective was to further develop the QI-ROI framework by exploring the categories of goals and benefits that reflect ROI from large-scale QI programmes. In other words, what QI authors and experts would deem or have deemed a return-on-investment from QI programmes. This knowledge was then used to compile types of benefits that if achieved, represent the QI-ROI. We also explored if and how QI benefits are linked to each other. The linkages were crucial in gaining insights into how the complexity of healthcare as well as QI as a complex intervention may impact ROI evaluation.

## Methods

### Underpinning theory

Our wider research project on the ROI concept is informed and underpinned by Complexity Theory. We deemed this theory pertinent, given the multiple QI objectives of multiple healthcare stakeholders. Complexity Theory encompasses a group of theories from different disciplines that highlight the interdependent, interconnected, and interrelated nature of a system i.e., human and technological components of an organisation [11, 37, 38]. These components influence each other in unpredictable ways with emergent consequences [11]. Therefore, complexity may lead to uncertainties, benefits, and challenges that may impact ROI. Various tools can be used to study this complexity in QI programmes [8, 39, 40]. However, in this study, Complexity Theory was used only to highlight the complexity during our analysis rather than to study it. We will examine this complexity in detail in our next study on ROI determinants.

### Review type

This paper is part of a larger Integrative Systematic Review on the ROI concept and its determinants from healthcare QI programmes. Our review is registered with PROSPERO, CRD42021236948. We have amended the protocol firstly to add additional authors as the complexity of the review called for more author perspectives. Secondly, we added the use of Framework analysis instead of Thematic analysis. A link to our PRISMA reporting checklist [25] is included in the supplementary files. We followed review guidance on Integrated Reviews by Whittemore and Knafl [41] and Conceptual Framework Development by Jabareen [42]. This led to 8 separate review stages. Stage 1; clarifying research question, involved background reading as is discussed in our protocol on PROSPERO. The remainder of the stages are reported here. Stages 2–3 involved searching and selecting literature. In stage 4 we assessed the quality of research studies, stages 5–8 are reported in the synthesis, analysis, and results sections below.

### Stage 2

#### Search strategy

We searched Medline, Embase, Global health, PsycInfo, EconLit, NHS EED, Web of Science, Google, Google scholar, organisational journals, as well hand-searched citations. Search terms were from these three categories: (1) healthcare or health*, (2) ROI related economic evaluation terms (SROI, CBA, CEA, CUA), as well as terms value, benefit, and outcomes, and (3) QI, and its specific methods. Table 1 contains definitions of search terms. No language/date limits were set to enable us to note any changes in QI-ROI conceptualisation over time. The search ended on January 30^th^, 2021. The search strategy is provided as Supplementary Table 1.

**Table 1:** Definitions of terms

Terms	Description
CEA	Cost-effectiveness analysis: Achieving more of the outcome for the same cost or achieving the same outcome for less cost, expressed in incremental benefits on Quality Adjusted Life Years (QALY), or incremental cost-effectiveness ratio (ICER)
CUA	Cost-utility analysis: Similar to CEA but for multiple outcome measures in quality-of-life units (QoL)
CBA	Cost–benefit analysis: Financial expression of costs and benefits from a programme in a cost–benefit ratio (CBR)
	CBA is the basis for ROI and SROI; CBA and SROI are societal perspectives, ROI is managerial/investor focused
ROI	Return on Investment: Expression of costs and benefits from a programme expressed in an ROI metric
SROI	Social Return on Investment: Expression of costs and benefits from a programme expressed in a ROI metric
	Includes benefits for society, environment and others. Engages various stakeholders in the calculation process
	Economic terms sources: [16, 43–45]
Value	Any outcome seen to be of importance, utility, or usefulness [46]
Benefit	Any outcome that produces useful, helpful, or advantageous outcomes (Cambridge Dictionary, 2022)
Outcome	A result or consequence of an action or process (Merriam Webster, 2022)
QI methods	Main QI methods include PDSA, Lean, Six-Sigma, Lean-Six Sigma, Audit & Feedback [47–49]
LARGE-SCALE QI	Programmes for whole or a large part of an organisation, or local, regional, national, or international collaboratives that combine clinical, strategic, workforce and organisational elements into a coherent quality improvement process to improve safety, capability, and capacity of an organisation [10, 50]
COLLABORATIVE	A QI collaborative (QIC) brings together multidisciplinary teams from different organisations and agencies to test solutions and share learning in a specific clinical or operational area [51]
HEALTHCARE ORGANISATION	(UK) A unique framework of authority within which a person or persons act or are designated to act towards some purpose as a direct provider of healthcare services (preventative, curative, rehabilitative, or palliative). Includes Local Authorities with Social care working in cooperation with the NHS [52]

#### Eligibility

**Fig. 1:**
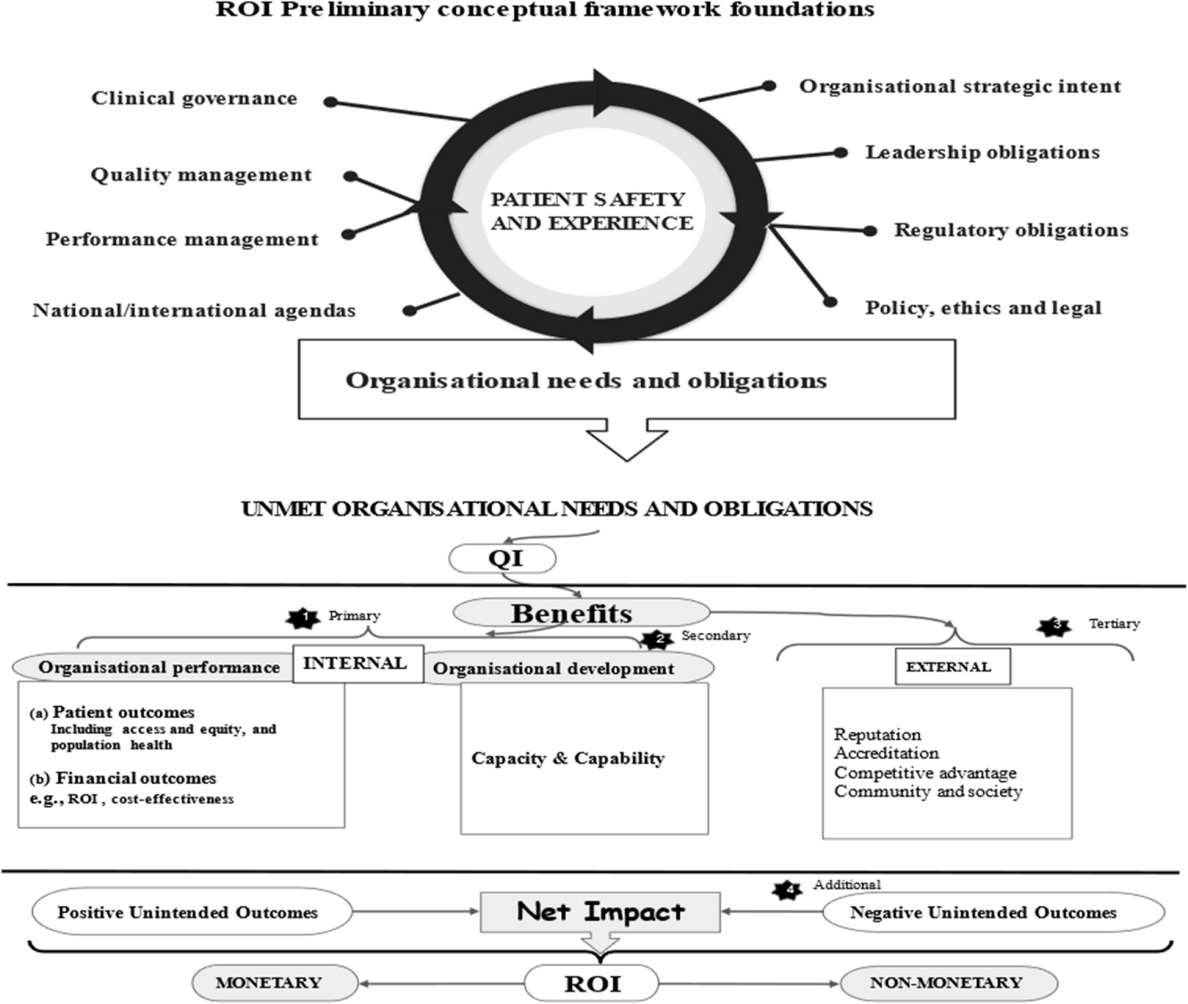
Preliminary QI ROI conceptual framework

During our initial search, many articles identified themselves as large-scale QI programmes. To focus our selection criteria, we developed a preliminary ROI conceptual framework (Fig. 1). This framework contained various needs and obligations of healthcare organisations [53, 54], which we assumed to signal desired organisational outcomes. The Framework had four criteria: 1) organisational performance (patients and financial outcomes), 2) organisational capacity and capability, 3) external relations (e.g., accreditation), and 4) unintended consequences (positive/negative). Organisational performance is a marker of how well organisations perform on delivering value for its stakeholders [55]. Thus, in a way it includes external relations, e.g., population health. However, they have been isolated here to deduce some unique external outcomes and obligations towards external stakeholders. We then used this framework to decide on eligibility. We included literature on discussions and evaluations of large-scale QI programmes at all healthcare levels (primary, secondary, tertiary) globally.

We included literature that mentioned at least three QI organisational goals or benefits, two of which had to be patient or financial outcomes. By doing this, we sought to isolate articles that discussed a wide range of QI outcomes, with patient and financial outcomes as basic organisational QI performance goals. In addition, articles had to mention use of at least one QI method and involvement of various stakeholders, in at least two organisational units. Altogether, this denoted a three-dimensional criteria: depth, breath, and complexity of programmes per organisation. Table 2 has Included/excluded article types.

**Table 2:** Eligibility criteria and selected article types

Eligibility	**Outcomes**	**ROI concepts**	**Level of analysis**
	QI Effectiveness or process outcomes e.g., goals achieved	Cost-effectiveness	Organisation
		Cost–benefit	
	QI economic outcomes e.g., savings	Value	
	Clinical outcomes e.g., symptoms	Benefits	
	Organisational outcomes e.g., development	QI outcomes/consequences	
	Short-term, intermediate, long-term, and impacts		**Type of literature**
			Empirical and non-empirical reports
			Conceptual and Grey literature
Included	**Large scale complexity, depth, and breadth**		
	At least one QI method used		
	At least three organisational outcomes		
	At least two organisational departments engaged		
Excluded	Articles where one department was engaged, two or less organisational outcomes were reported, and pre-prints		

### Stage 3

#### Screening and selection of articles

Citations were downloaded onto Endnote, Clarivate [56] to compile a list of citations and remove duplicates. Rayyan software [57] was used to screen abstracts and full titles as per our search criteria. Screening and selection were performed by two independent reviewers, ST and MM. To refine our selection criteria, five articles were initially selected and discussed to clarify any uncertainties. The two reviewers then completed the screening and selection of the remaining articles independently: ST 100%, MM 5%. Overall agreement was over 90%. Disagreements were discussed and settled by ST and MM, as well as with co-author CH.

#### Data extraction

Data extraction was performed using words and phrases in the preliminary conceptual framework as well as outcomes in the review’s search terms. We searched for these from all parts of an article where QI benefits, outcomes, and goals may be discussed. This included the introduction, aims, objectives, results as well as discussion and conclusion. Articles were tabulated according to type of article, country, setting, programme type, and outcomes discussed. Data extraction file has been included as Supplementary Table 2.

### Stage 4

#### Quality assessment

For researchers of integrative reviews and conceptual development, quality assessment is optional as the quality of studies has little or no bearing on concept development [41, 42]. As such, there was no intention to exclude articles based on their quality. However, to understand the scientific context in which QI benefits are discussed, we assessed all empirical studies using specific quality assessment and reporting tools. For reviews, we used Critical Appraisal Skills Programme (CASP) [58], for mixed methods, the Mixed Methods Appraisal Tool (MMAT) [59], for implementation studies; Standards for Reporting Implementation Studies (STaRI) [60]. For economic evaluations, the Consolidated Health Economic Evaluation Reporting Standards (CHEERS) [61], and for QI, the Standards for QUality Improvement Reporting Excellence (SQUIRE) [62]. As these are different tools, there was no single criteria to judge collective study quality. We therefore assessed the number of appropriate items reported or addressed as per respective study’s tool. We assigned good if 80–100% items were addressed, moderate if 50–79% of items were addressed, and poor if less than 50%.

### Stages 5–7

#### Data integration, synthesis, and analysis

We followed Framework Analysis [63], using guidance by Braun & Clarke [64] thematic analysis, and deductive-inductive hybrid analysis by Fereday & Muir-Cochrane [65]. This allowed us to identify data from our ROI preliminary conceptual framework as well as any emerging data related to ROI. During the synthesis we summarised findings from the integrated literature and compiled a table of themes, sub-themes, and related outcomes. In the analysis, we noted the complexity and relationships between these themes and outcomes.

The result was a developed QI-ROI framework that outlines the ROI concepts from our first study (e.g., efficiency, productivity, cost-management, cost-saving). Productivity is the quantity of outputs/returns (e.g., patients seen) per investment/input (e.g., staff). Efficiency is achieving those outputs from same or less inputs with least or no waste (e.g., in time, money, effort) [66]. Cost management are certain strategies used to manage cost [67]. Cost saving can be an outcome of efficiency, productivity, and cost-management. This initial QI-ROI framework was combined with the categories of QI benefits from the current study to form an extended QI-ROI framework.

### Stage 8

#### Results

**Fig. 2:**
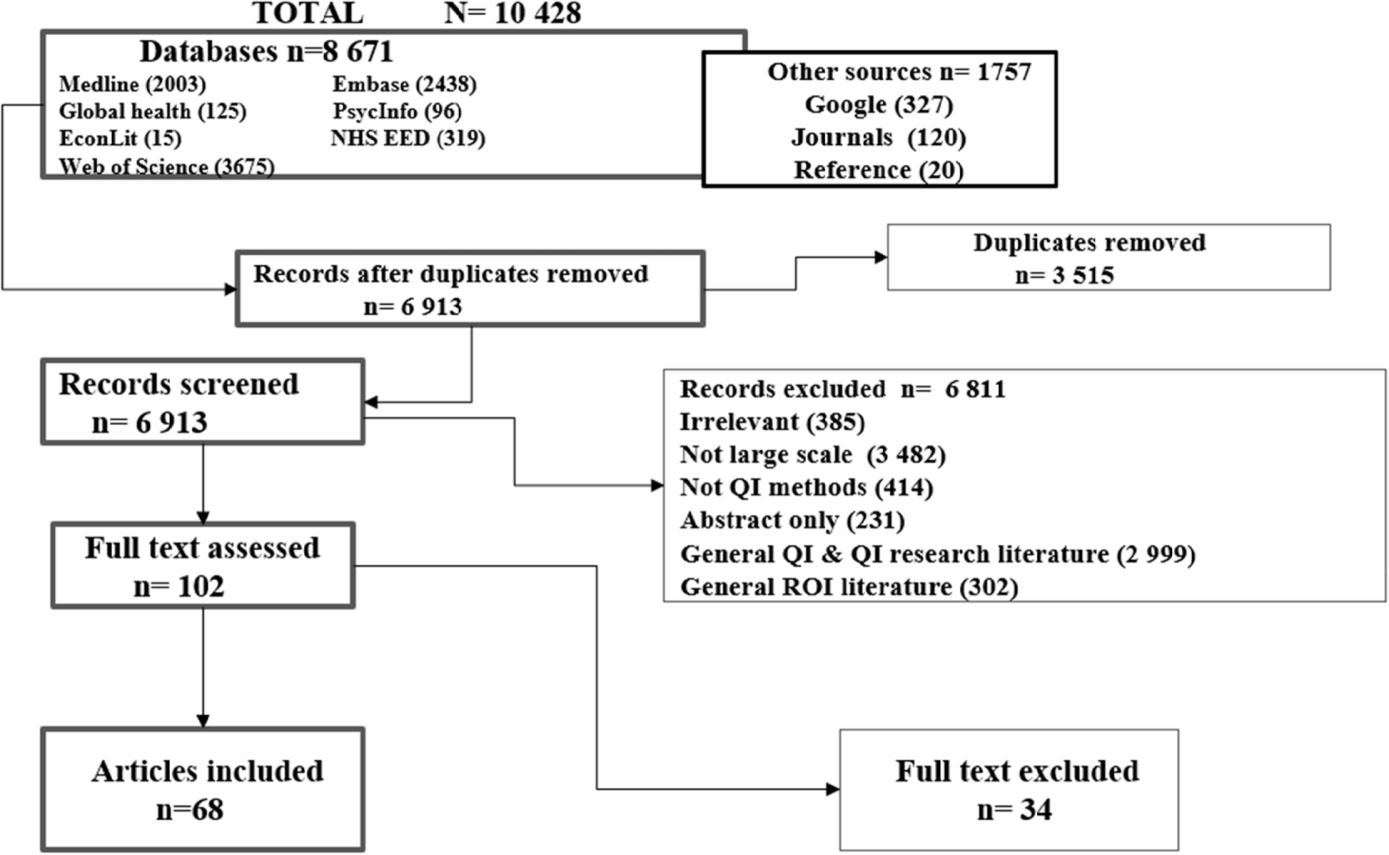
PRISMA flow chart

A total of 10 428 articles were retrieved, 10 327 were excluded for various reason as shown in Fig. 2. One hundred and two (102) articles were eligible, 34 were excluded and 68 included. Included articles were: Conceptual *n* = 24, Quantitative studies *n* = 19**,** Qualitative studies *n* = 3, Mixed-Methods studies *n* = 8, Systematic Reviews *n* = 8, Literature reviews *n* = 2, Brief Reports *n* = 4. Together, the articles represent 18 years of QI evaluation (2002–2020). Excluded articles were where programmes engaged a single department and/or discussed two or fewer QI outcomes/goals. Thirteen of these were collaboratives. There was one pre-print. A link to the excluded studies document is available in the supplementary files.

##### Article characteristics

Included articles covered different healthcare levels and disciplines. Primary care included public health, child and maternal health, and mental health. Secondary and tertiary healthcare included mental health, medical and surgical care, critical care, accident and emergency and acute care services, paediatrics and neonatal care, outpatients, pharmacy, and laboratories. One article covered both health and social care, and another article was about QI in a healthcare related charitable organisation. Articles were from these global regions: Africa, Asia, Europe, Australia, and Canada. The mostly represented regions were the US and the UK. Only 15 of 68 articles were economically focused. ROI was a specific subject of only four articles [68–71], and five authors discussed development of QI business cases [33, 72–75]. One article discussed cost–benefit analysis from a qualitative perspective [76], there were two economic systematic reviews, and three economic evaluations. de la Perrelle et al. [77] also found this lack of economic evaluations in their systematic review. However some authors reported their implementation costs [78–80]. The summary of included studies can be found as Supplementary Table 3.

##### Quality of studies

Thirty articles were not subject to quality assessment. These were conceptual articles, unsystematic literature reviews, and brief reports. Thirty-eight articles were subjected to quality assessment: 19 quantitative studies, three qualitative studies, eight mixed-methods studies, and eight systematic reviews. Of the 38 studies, 39% reported or addressed 80%-100% of all items required, 43% reported on 50%-79% of the data required, and 18% reported below 50% of items required by their respective reporting tool. The main areas of poor rigour were: ethics (29%), statistical analysis methods (75%), discussion and management of study limitations (42%). For some mixed methods studies (29%), integration of quantitative and qualitative data was unclear. In some cases, these issues may merely reflect poor reporting. However in the absence of data, poor rigour was assumed. Overall, the quality of the studies was summed-up as moderate. The quality assessment summary is provided as Supplementary Table 4.

##### Data synthesis and analysis

Authors either directly studied QI outcomes, reported additional QI outcomes and benefits, and or discussed QI goals and missed opportunities. A number of papers reported financial savings or had savings as a goal [77, 81–88]. Gandjour & Lauterbach [89] noted that cost-saving was more likely when improving an over-use or misuse problem. For example, an article reported cost-reduction from malpractice suits [74]. Financial benefits through QI were mostly internal to organisations, and a small number involved societies and healthcare funders [73, 75].

There was a shared view that quality and patient safety should be more central to QI and investment goals than financial outcomes [72, 88, 90–95]. This view had not changed over time. Thus, QI goals were primarily improving patient outcomes through systems, structural, process, and behavioural improvements. This enabled improved efficiency and productivity. Efficiency and productivity enabled managers’ abilities to manage, minimise, or reduce costs, and eventually save costs [73, 94, 96–98]. Systems efficiency helped improve staff efficiency, effectiveness, productivity, and experience, which benefited patients [84, 99, 100]. Improved systems enabled improved organisational capacity, capability, and resilience [93, 101–106].

Most authors highlighted that good quality and patient safety relied upon good staff outcomes and leadership. A few studies focused on some of these specific areas. Examples include Mery et al. [71] who studied QI programmes as an organisational capability and capacity development tool. Hatcher [83] studied QI as a staff safety promotion tool, Lavoie-Tremblay et al. [99] evaluated QI as a tool for team effectiveness. Furukawa et al. [107] and Heitmiller et al. [84] focused QI towards environment sustainability. MacVane [96] saw QI as a governance tool. Williams et al. [100] focused on both staff and patient outcomes. QI was also used to operationalise organisations’ strategies [93, 108]. Staines et al. [108] found that a positive QI reputation allowed recruitment of a suitable CEO.

There was a general recognition that QI does not always achieve its intended goals. Additionally, some QI strategies were more successful than others [80]. Particularly, some literature reviews and empirical studies reported variable, mixed, or inconclusive results [86, 109–115], even a decline in quality [99]. A few articles discussed negative unintended outcomes [81, 100, 104, 110, 112, 114, 116–119]. de la Perrelle et al. [77] noted this lack of reporting of negative findings in their review. They suspected this to be due to publication bias. Rationales for not achieving goals were given as implementation difficulties related to contextual and behavioural challenges [78, 114, 120, 121].

Some authors noted that overall benefits accrued over time during phases of a programme’s implementation process [80, 122]. Morganti et al. [123] noted different measures of QI success but suggested that spread of a programme was a measure of lasting success. Sustainability of outcomes was therefore also seen as an important achievement by most authors. This was supported by some of the literature which also indicated that successful QI built legacies mainly through spreading, embedding, and sustaining improvements [78, 93, 101–106]. This finding was confirmed by impact studies, extensive QI programme evaluations and discussions of overall QI impacts [69, 85, 87, 93, 103–106, 108, 115, 116, 119, 121, 124–126]. These literatures elaborated on QI goals, failures, and successes, as well as the lessons learnt. Authors suggested that lessons and cultural changes as a result of QI were essential to meeting patient safety needs [93, 109]. Authors highlighted that ultimately, QI benefited a wide range of stakeholders at different levels in different ways.

##### Themes

Based on the findings, we compiled data into four overarching themes (Table 3). These themes aligned with our ROI preliminary framework; however, adjustments were made to reflect the findings. Organisational capacity and capability was renamed organisational development to acknowledge the broader organisational outcomes. This included all the outcomes that develop and improve organisations’ ability to fulfil their duties. Resilience and QI legacy were additional sub-themes under organisational development. External relations was renamed external outcomes to reflect the broad outcomes beyond relationships with regulators, communities, and other organisations. External outcomes were extended to include collaboration, societal and environmental outcomes, and incentives. Incentives included accreditation, awards, ranking, competitiveness, influence, power, and financial rewards.

**Table 3:** Themes and associated outcomes

ORGANISATIONAL PERFORMANCE			
Theme	Sub-themes and associated outcomes	Sources	Exemplar quotes
Patient outcomes	● Clinical outcomes ● Patient safety ● Patient engagement ● Patient empowerment ● Patient experience ● Socio-economic benefits ● Service user recruitment	[33, 68–135]	Clinical outcomes
			“The adverse event rate increased from 2.9 to 4.8 per 100 patients in control hospitals and declined from 6.2 to 3.7 among SPI1 hospitals”. Authors; Benning et al. (2011, p. 11)
			Patient experience
			“…improving process performance, including waiting time reduction and patient flow with the subsequent impact of increasing patient satisfaction”. Authors; Honda et al. (2018, p. 70)
			Social impacts
			“…the list of possible social returns … became quite long, and each social impact (for example, less patient time spent in hospitals) could cascade into broader social impacts (for example, increased productivity, increased efficiency at hospitals, benefits of expenditures in other areas …)”. Authors, Moody et al. (2015, p. 30)
Financial outcomes	● Cost saving ● Revenue generation ● Cost-management ● Cost reduction ● Cost avoidance ● Financial stability	[33, 68–71, 74, 76–79, 81, 84, 86, 88, 92, 93, 108, 110, 116, 119, 124, 130, 134]	Legal costs reduction
			“In the last 6 years our professional liability exposure has decreased. It is possible that this resulted from higher quality care”. Authors; Swensen et al. (2013, p. 47)
			Cost reduction and revenue generation
			“The large-scale QI …has the potential for ROI at multiple levels… opportunity to improve efficiency, remove waste, lower cost, and increase revenue.” Authors; O’Sullivan et al. (2020, p. 3)
ORGANISATIONAL DEVELOPMENT			
Strategic goals	● Achievement of organisational strategies ● Improved alignment with strategies: refinement and clarification ● Generation of organisational mission, objectives, and priorities ● Improvement in organisational ethical, moral, legal, and value obligations ● Creating new personal and meaningful operating models ● Patient-centredness ● Staff-centredness ● Decision-making and problem-solving improvement ● Overall organisational performance improvement	[69, 73, 77–79, 81, 82, 87, 94, 97, 102, 104, 105, 111, 117, 121]	Increased market share
			“Significant improvements in waiting time and number of new patients were identified for two of the interventions”. Authors; de la Perrelle et al. (2020, p. 5)
			Strategy to engage service users
			“…to improve the total quality of every service user’s journey throughout the mental health system… by developing the capacity and skills of local care communities in order to make fundamental improvements in the way services are provided” Participant; Worrall et al. (2008, p.13)
			“At a policy level, patient safety is now articulated as a clear priority and has become more closely linked with the national drive to improve quality of care while increasing productivity and efficiency” Authors; The Health Foundation (2011, p. 27)
Governance	Improve organisational transparency, accountability ● Improving clinical effectiveness and patient safety ● Improving human resource effectiveness ● Risk management ● Compliance with performance criteria ● Performance management and measurement beyond clinical governance to organisational governance	[81, 90, 96, 102, 123, 132]	“We are currently exploring, through early pilot projects, a range of board development interventions and improvement approaches, to enable better governance of patient safety within organisation”. Authors, The Health Foundation (2011, p. 26)
			“This flexibility and enabling grassroots practitioners to become the problem solvers is the key to changing over to a lean management or governance system”. Authors; MacVane (2019, p. 84)
Human resource development	Improved staff capabilities ● Raising awareness on QI methods, patient safety, inefficiencies, and costs, ● Increase staff ability to assess which problems were best suited to QI ● Improved personal and career development and job security ● Staff engagement ● Staff empowered Improved staff experience ● Improved motivation, and enthusiasm, Improved staff capacity ● Supporting recruitment and retention, ● Improved job security, and reduced staff sickness ● Developing new QI roles ● Role clarification	[73, 74, 79, 81, 89, 95, 97, 99, 101, 103, 104, 106, 107, 113, 117, 124, 125, 129, 134]	Staff capabilities
			“Ninety-one per cent felt the Collaborative had empowered them to make a difference in reducing the number of pressure ulcers. Feedback given from one of the two people who did not answer this way stated that it was ‘already part of job role.’” Authors, Wood et al. (2014, p. 6)
			“…staff reported benefits to the social and work environment, but perhaps most significantly working on the programme was described by some staff as a long awaited opportunity for personal or career development” Authors; Morrow et al. (2012, p. 248)
			Staff experience
			“Greater knowledge tended to produce greater enthusiasm” Authors, The Health Foundation (2011, p. 11)
			“As great as the financial impact of purchasing safety devices and of a needlestick injury may be, the nonfinancial impact can be even greater. We desire the work environment to be as safe as possible for our staff”. Authors; Hatcher (2002, p. 413)
			Staff capacity
			“The apparent improvement in staff sickness rates; or the recorded decrease in bed numbers apparently associated with the trust’s analyses showing reduced length of stay on the targeted wards”. Authors; Hunter et al. (2014, p. 64)
			“…the programme appeals to the intrinsic values of frontline (particularly nursing) staff and has had a positive impact (key themes were: equipping staff with new skills, more time for better care, improved patient experiences, cost savings, and higher staff satisfaction and retention”. Authors; NHS Institute (2011, p. 17)
Process, structural, and systems	Efficiency and productivity ● Team efficiency ● Systems efficiency ● Processes efficiency Resource management ● Optimisation, or leveraging of existing systems ● Facilitating effective resource allocation ● Spreading of costs and benefits or off-setting other organisational benefits Structural changes ● Guiding patient safety infrastructure development ● Reduction of incidences of violence	[33, 71, 73, 75, 79, 81, 84, 87, 91, 93, 95, 110, 114, 119, 121, 132, 135]	Process improvement
			“Process mapping the care of patients with sepsis, presenting key issues visually and as a gap analysis were essential to identify the core elements of the clinical pathway, to introduce structural changes”. Authors; Thursky et al. (2018, p. 7)
			Resource management
			“…this made it possible to revise the procedure for filing and monitoring patient files by nurses, thus reducing the time allocated to this activity by one hour per week.” Authors; Comtois et al. (2013, p. 174)
			“The collaborative learning process during audit and feedback, to enable self-monitoring and provision of action plans, resulted in various institutional changes…” Authors; Brink et al. (2017, p. 1232)
			Structural improvements
			“Benefits included better organised working environments, fewer patient safety incidents, and cash savings in terms of returned excess stock”. Authors; Morrow et al. (2012, p. 246)
Culture and climate	Developing a QI safety culture ● Culture aligned to people ● An organisational learning culture ● Change from performance and regulation to continuous improvement ● Change from project orientation to capacity and capability building ● Change from top-down to bottom-up development ● Culture of shared leadership models ● Culture of collaboration ● Flexible and inclusive culture ● Challenging of existing mental models Improved organisational climate	[33, 95, 101–103, 105, 117, 119, 125]	Culture
			“… I don’t think you can buy the attitude and mental approach that needed to happen. And I truly think money and resources wouldn’t have helped. …I think that is the level at which the intervention to change the system should have been, right at a deeper level. Not resource, not environment, but more the deep cultural partnership interpersonal level” (p. 103). Participant; Worrall et al. (2008, p. 103)
			“In those trusts we have rated as outstanding; we have found a culture of quality improvement embedded throughout the organisation.”. Authors; CQC (2018, p. 2)
			Climate
			“There were also significant improvements in secondary outcomes: patients’ overall rating of ward quality; nurses’ positive affect and team climate”. Authors; Williams et al., 2020, (p. 45)
Leadership development	● Leadership development ● Leadership effectiveness	[74, 92, 96, 102, 103, 107, 108, 116, 117, 120, 121, 127, 128]	Leadership development
			“…relatively junior staff with limited practical experience are now running the collaboratives. Without the right leaders, there is a risk that collaboratives are pale imitations of effective programmes”. Authors; Collins and Fenney (2019, p. 18)
			Leadership effectiveness
			“Having been involved in some major NHS improvement collaboratives, including one looking at adverse drug events, I initiated an internal collaborative on medication error”. Participant; The Health Foundation article (2011, p. 20)
Internal collaboration	● Intra-organisational learning networks ● Team-working ● Team cohesion ● Enhanced communication	[33, 83, 98, 102, 103, 108, 121, 122, 131]	Team-working
			“…the process successfully facilitated a welcome shift from a ‘parent–child’ relationship where the pharmacists are always seeking the junior doctors and pointing out mistakes that need to be amended to a more effective and efficient ‘team work’ approach where junior doctors and clinical pharmacists work together to generate a safe discharge…” Authors; Botros and Dunn (2019, p.8)
Research development	● Increased awareness of QI evidence-base enhancement ● Stimulating ideas on innovative research methods development ● Evidence dissemination ● Increased focus on financial outcomes	[83, 95, 96, 103, 113, 119, 121]	“The three strands of evaluation of the Safer Patients Initiative have surfaced some important reflections on research and evaluation of complex, organisational interventions”. Authors, The Health Foundation (2011, p. 23)
			“A program called “Measurement for Management,” offered by Qulturum with IHI input28and open to teams from across Sweden, was created following the 2006 study, to help participants build system-level capacity for measurement, data collection, and interpretation”. Authors; Staines et al. (2015, p. 26)
Innovation	● Development of new ways of working ● Development of new tools and methods	[85, 94, 102, 112, 113, 128, 133]	“NHS Safety Thermometer data collection tool was developed by the national programme team during the design period of phase I and refined iteratively thereafter”. Authors; Power et al. (2016, p. 9)
IT development & data management	● Improved data management ● local ownership of data monitoring and reporting, ● Data transparency and sharing, ● Data used to guide improvements	[33, 79, 93, 96, 102, 103, 107, 108, 115, 121, 127, 128, 135]	“... the data collection before and what we collected data on afterwards were different things really in a way. So they had to be retrospective to get some of the baseline stuff, because we didn’t know what was going to come out and the changes that were going to happen.” Participant; Hunter et al. (2014, p. 62)
			The QI activities often resulted in an improved understanding that measurement was an important part of any Method adopted. In addition, staff often also realised that suitable metrics were not available, or that the data were of poor quality”. Authors; Hunter et al. (2014, p. 81)
			“Ownership of our data and ownership, that’s one of the things that’s really improved the clinical team I think”. Authors; Worrall (2008, p. 120)
QI legacy	● Sustainable benefits from previous programmes ● Created new standards and expectations of care ● Increased collective QI knowledge and skills ● Financial sustainability ● Performance sustainability ● Sustained organisational capabilities ● QI legacy through implementation outcomes spread or scale-up ● Built foundations for bigger more complex programmes ● Increased capacity to learn from challenges, failures and successes of self and others	[71, 74, 75, 81, 92, 94, 102, 104, 107, 114, 119, 121, 123, 124, 127, 128, 131, 133]	“Throughout five years since implementation of MEWS‐Sepsis tool patient screening, the organization has realized a sustained decline in sepsis mortality of 24%” Authors; Roney et al. (2016, p.3)
			They also provide the bedrock for future improvement in the quality, safety and efficiency of integrated hospital and community services, as well as between adult social care, mental and physical health care, and acute and long-term services.” Authors; Pearson et al. (2017, p. 5)
			“…we found that staff continued to apply these principles to their QI work even as organisational contexts changed over time”. Authors; Robert et al. (2020, p. 38)
			“I think that the legacy of MHIP and the restructuring has meant that we really have taken a much more defined systems approach, and I think much better clarity about roles and responsibilities and accountability in the system”. Participant; Worrall et al. (2008, p. 118)
Organisational resilience	● Achievement of a high reliability, high performing, and self-sustaining organisation ● Coping with changing and unstable contexts ● Organisational learning	[71, 92, 102, 128]	“Projects can fail to show improvement or fail to sustain themselves. ELFT are interested in such cases too, and the considerable learning they can yield. This interest in failed projects, and difficult to improve areas, sends the message to staff that all is not lost if results are limited” Authors; O’Sullivan et al. (2020, p. 6)
EXTERNAL OUTCOMES (MACRO)			
Incentives	● Recognition as a leader and influencer ● Financial incentives, awards, accreditation, ● Improved competitiveness, ● Improved influence and power ● Positive reputation ● Pride for the organisation and staff ● Improved bargaining power ● Accreditation ● Reduced regulation and oversight	[33, 77–79, 85, 91–93, 96, 100, 103, 107, 108, 116, 127, 128]	Influence
			“Although the Safer Patients Initiative did not achieve the level of organisational impact hoped for within the timeframe of the programme, it did have a significant effect and influence on participating hospitals and their staff, on patient care and on the wider NHS system”. Authors; The Health Foundation (2011, p. 14)
			Awards
			“This RPIW was frequently mentioned by interviewees as an exemplar that demonstrated the positive benefits of the NETS programme. It received national recognition through the Health Service Journal awards”. Authors; Hunter et al., (2014, p. 63)
External obligations	● Compliance with oversight, accreditation, regulation	[33, 100, 103, 115, 135]	“Holding providers accountable for blood product wastage contributed to the waste reduction and could be used as a component of the provider’s ongoing performance profile, which has recently become a Joint Commission requirement”. Authors; Heitmiller et al. (2010, p. 1895)
			“Most of the NHS trusts in England that have been given an outstanding CQC rating have implemented an organisation-wide improvement programme”. Authors; Jones et al. (2019, p. 6)
Community and society benefits	● Community engagement ● Improved community resources ● Support for carers, children, and families ● Socio-economic benefits	[33, 68–70, 78, 88, 92, 107, 111, 113, 120, 129, 134]	External benefits
			“The greatest benefit from these 6- to 9-month QI projects was internal, yet the communities also reaped significant external benefits”. Authors; Crawley-Stout et al. (2016, p. E35)
External collaboration	● Data sharing ● Shared governance ● Multi-stakeholder engagement and alignment ● Foundations and maintenance of strategic relationships ● Long-term learning networks ● Improved multi-organisational relations ● Development of deeper awareness of collective issues.	[69, 72, 84, 87, 92, 93, 98, 103, 107, 112, 115, 119, 126–128]	Improved organisational relations
			“There was also a local history of difficult relations between hospital and community services. Service reconfigurations that maintain stability against such a backdrop and which lead to important signals of improvement are a success. They also provide the bedrock for future improvement in the quality, safety and efficiency of integrated” Authors; Pearson et al. (2017, p. 5)
			Shared Governance
			“Opportunities to train with other NHS NE organisations, to jointly redesign pathways and to speak the same language of improvement, were highly valued”. Authors; Hunter et al. (2014, p. 74)
UNINTENDED OUTCOMES ((MICRO, MESO)			
Positive unintended outcomes	● Gaining new insights on related organisational needs ● Improvements in untargeted departments or patients ● Incidental innovations ● Enabling communication ● Enabling targeted recruitment of QI staff and leaders ● Academic development through creation of patient safety or QI training ● Learning from failure and negative outcomes	[70, 77–79, 85, 107, 108, 122, 130, 131]	Incidental innovations
			“A multidisciplinary team with existing expertise in tracheostomy care commenced detailed tracheostomy ward rounds, providing a different context to the other sites. Local MDT oversight teams were established at all sites…” Authors; McGrath et al. (2017, p. 7)
			Enabling communication
			“The attention paid to Patient Safety had been a door opener. Patient Safety made it possible for hospital CEOs to discuss accountability with physician”. Authors; Staines et al. (2015, p. 25)
Negative unintended outcomes	‘Top-down distortions’ ● Scepticism about focusing too narrowly on managerial goals ● QI exhaustion; QI ‘constant hammering’ demands ● Perceived loss of autonomy ● Feeling bullied or intimidated ● System gaming or manipulation ● “top-down distortions” (Robert et al. 2020, p. 39) External imposition ● Side-lined local goals in favour or external goals High resource demands ● Increased need for support for staff and leadership ● Increased financial resource needs ● Data burden due to the data demands from multiple stakeholders ● Increased resource and staff costs Duplication ● Duplication of resource needs, QI tools, and methods due to top-down and or external QI goals Loss of revenue ● Reduced patient enrolment as a service was no longer needed or needed less Loss of buy-in ● Disengagement, ● Loss of enthusiasm and motivation ● Staff disillusionment	[79, 80, 82, 88, 94, 95, 110, 112, 113, 116–121, 124, 127, 128]	‘Top-down distortions’
			“…top-down distortions through performance management systems”, which caused a shift away from a longer- term vision of empowering ward teams to take ownership, potentially limiting positive long- term legacies. Participant; Robert et al., (2020, p. 39)
			“There’s a constant hammering, it’s almost like a squeaky wheel. I wouldn’t call that performance management, in effect it comes down to a set of KPIs for the system and everything that is perceived to improve that, gets pushed”. Participant; Masso et al. (2010, p.356)
			External imposition
			“…they also expressed concern about aspects of the oversight of collaboratives, including the pressure to deliver complex programmes and demonstrate benefits within very short timescales, and the amount of time small teams needed to dedicate to the process of justifying their collaborative’s effectiveness”. Authors; Collin & Fenney (2011, p. 22)
			High resource demands
			“To bring about large-scale improvement is far costlier than anybody ever envisaged and unless you really are willing to make the true investment you often don’t get the sustained change improvement that you require”. Participant; Worral et al. (2008, p. 101)
			“Only one quarter of all respondents believed that training had been sufficient and 18% felt that resources had been sufficient to implement Lean”. Authors; Goodridge et al. (2018, p. 18)
			The challenges this posed for QI leads must not be underestimated, with the burden of collecting data (for NELA and ostensibly for use as part of the EPOCH improvement work) may have overwhelmed many”. Authors; Stephen’s et al. (2018, p. 11)
			Duplication
			“…interviews revealed some anger among informants. The disappointment was the highest in Primary care, as this sector already had a clinical information system in the past”. Authors; Staines et al., (2015, p. 25)
			Loss of revenue
			“a negative return on investment for the program in the short run. In the longer run, a positive return would occur through avoidance of increased morbidity, but, because of enrolee turnover, both organizations might not be able to realize that return”. Leatherman et al. (2003, p. 21)
			Loss of buy-in
			“…ward staff generally did not feel as engaged in the work and medical engagement remained one of the programme’s biggest barriers”. Authors; NHS Foundation (2011, p. 20)
			“Nurses who have been previously captured by the panacea of being ‘productive’ and ‘releasing time to care’ may simply have escaped the captivity and control of that dreamlike desire, and are just refusing to engage with the dance of efficiency (Rudge 2013) in White et al. (2014, p. 2420)

Negative unintended outcomes include any negative impact resulting from a QI programme. These were external imposition, top-down distortions, duplication, high resource demands, loss of revenue, and loss of buy-in. Authors reported that at times external or managerial agendas were superimposed over other QI goals [108, 116, 127, 128]. At times this caused duplication of processes (e.g., data collection) and or increased demand on already stretched services. In addition, successful QI can cause loss of funding as services become absolute [108]. Eventually different negative outcomes may cause staff or leaders to disengage from current or future QI.

Positive unintended outcomes were difficult to delineate as often programmes were geared towards patient outcomes but impacted other parts of an organisation in the process. However, as improvement strategies involved changing systems and human behaviours, improvement of these aspects must be intended. We therefore had this sub-theme only include new innovations and opportunities. The final overarching themes were named 1) organisational performance (two sub-themes), 2) organisational development (12 sub-themes), 3) external outcomes (five sub-themes), 4) unintended outcomes (two sub-themes).

**Fig. 3:**
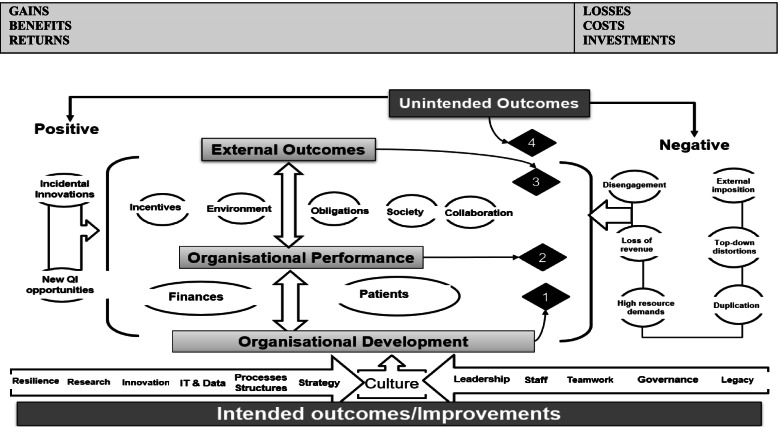
Updated preliminary ROI Conceptual Framework. Most QI goals and outcomes affect an organisation’s culture. The four overarching themes are connected and influence one another e.g., improved performance enabled attainment of external incentives. An overlap exists amongst these themes, e.g., collaboration was improved both internally (organisational development, and externally as an external QI benefit)

Based on the themes, we updated our ROI preliminary conceptual framework to map the four overarching themes that represent QI-ROI (Fig. 3). The beneficial outcomes are presented under the headings “gains, benefits, returns”, whilst negative outcomes are presented as “losses, costs, investments”. These concepts are technically defined differently. They are used together here to denote their co-existence within QI programmes. For example, loss of revenue is a potential investment lost, high resource demands may require investment or incur costs, duplication is inefficient and costly, loss of buy-in is a costly setback. All will raise money spent or lost if not well managed or avoided. They may also affect organisational performance and development, as well as stakeholder engagement in future programmes. Thus, impacts are both monetary and non-monetary.

Authors also saw investments as both in monetary and non-monetary forms. These were viewed as both equally essential for patient safety and quality. Some of these investments were part of ongoing organisational strategies. Investments included staff time, recruitment and retention costs, training costs, patient engagement costs [68, 69, 77, 95, 108, 113, 114, 116]. Some investments depended on the goodwill of the staff and patients and were seen as priceless [119]. Staines et al. [108] referred to two types of investments: “hard” infrastructure (e.g., technology) and “soft” infrastructure (e.g., awareness, commitment, and culture).

The literature also noted that QI outcomes are interlinked and interrelated, and as such QI-ROI may not be readily observable. Deducing ROI may require studying “cause-and-effect chains” [92] (p. 2) or an ROI chain; the link between events from a given investment to a given outcome. Sibthorpe et al. [113] saw this as important for understanding QI impacts and attracting QI investment. This can be done by tracking inputs, processes, outputs, and outcomes as much as possible throughout a programme. By doing this, the integrity of the ROI chain may be assessed by identifying areas where QI-ROI is created, lost, or influenced. This may then help maximise QI-ROI. However, tracking this chain in complex contexts may be a challenge.

##### The QI-ROI chain

In complex systems, programme inputs, processes, outputs are not a once-only event, occurring only at initial implementation. Outcomes of earlier inputs, outputs, and processes become inputs in the next phase and so forth until the final impact is achieved (end-ROI). It may therefore be helpful to recognise and celebrate earlier achievements [33, 97]. Further, before a final impact is realised, a programme may act and interact with several variables. Due to this complexity, the linkages may resemble a web rather than a chain. The literature attested to the fact that QI impacts are unpredictable, and difficult to measure [33, 113, 119]. QI inputs may or may not be converted into active QI ingredients that will affect organisational change and improvement [80]. For example, if one of the strategies is to train staff; do they actually learn what is needed? The answer would depend on several internal and external determining factors [78, 79, 114, 120, 121]. Such factors may force adaptations, influence fidelity to strategies, sustainability, and decisions to proceed, de-implement or disinvest.

**Fig. 4:**
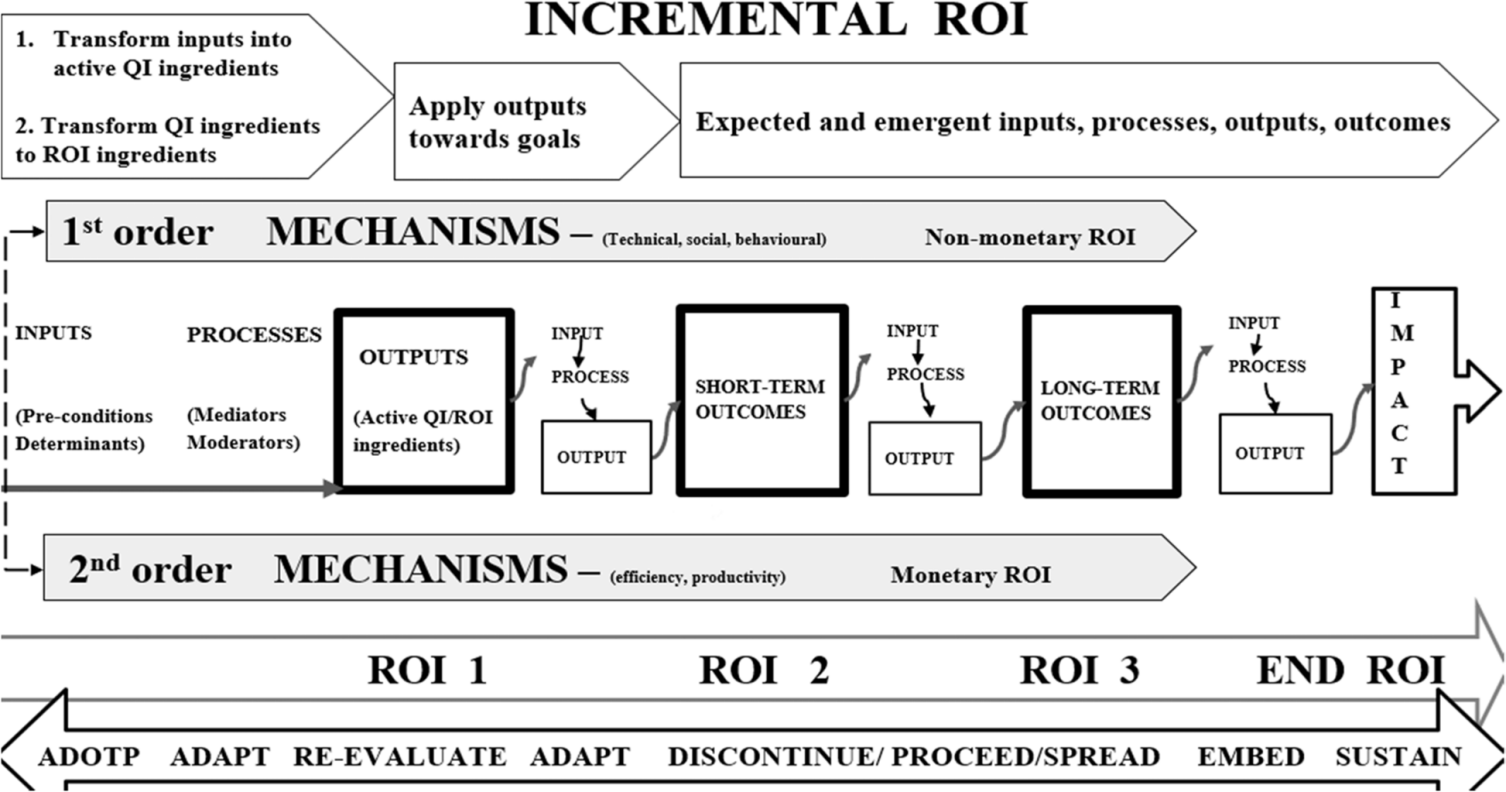
QI-ROI Chain

**Fig. 5:**
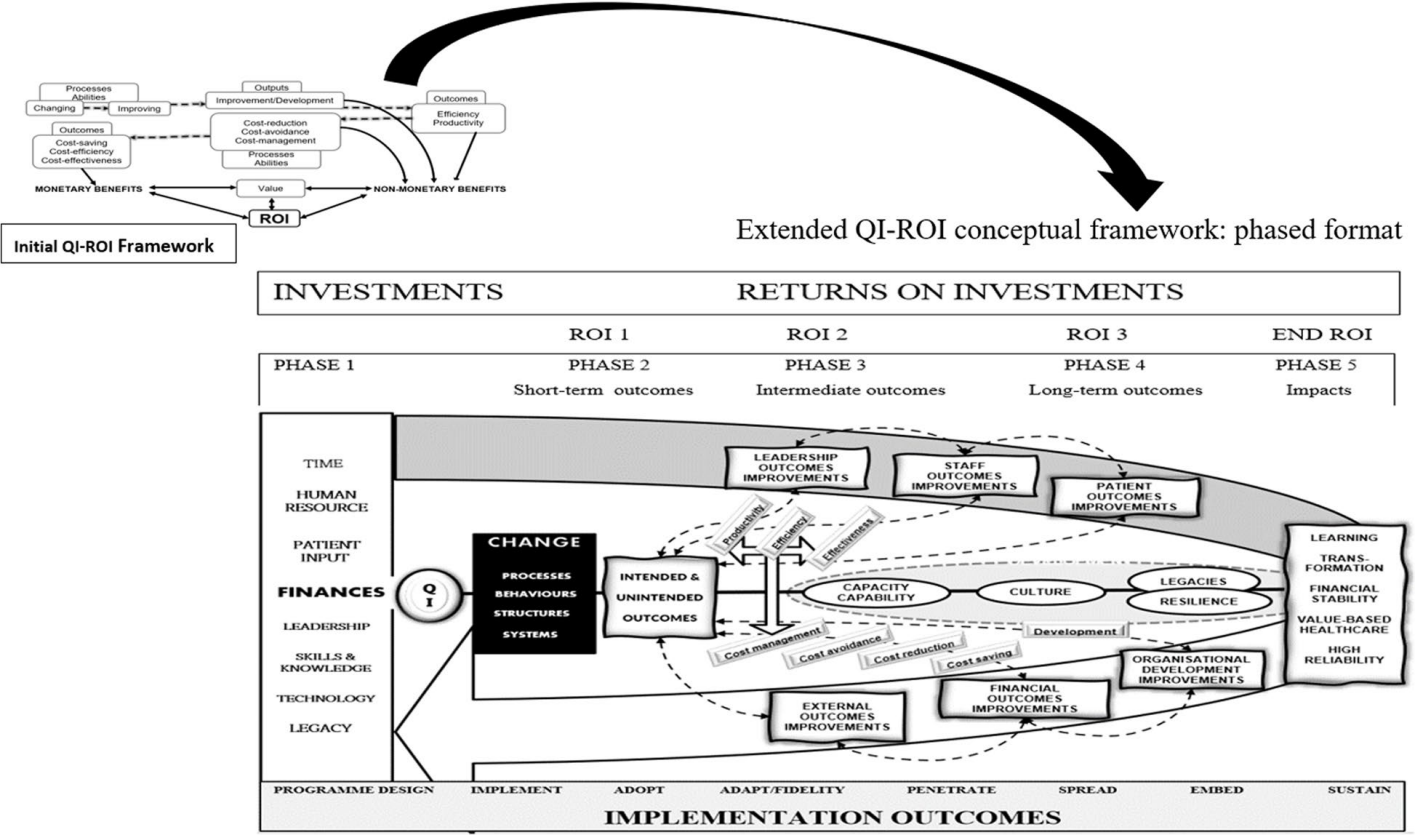
Extended QI-ROI conceptual framework: phased format

The ROI chain is illustrated here in Figs. 4 and 5. Figure 4 demonstrates that the overall ROI results from changes in processes, structures, and systems. These may be visible through behavioural (human and systems), and technological improvements, before final impact and ROI can be detected. Two-tier order mechanisms are alluded to here; the first order mechanisms operationalise QI strategies and provide non-monetary ROI, whilst the second order mechanisms convert QI efforts into financial returns. A first order mechanisms may be for example increased staff proficiency leading staff development, whilst a second order may be improved productivity due to increased proficiency. Productivity may then help save costs.

In summary, different investments are made towards a QI programme and a change is propagated through changing and improving processes, behaviours, systems, and structures. Technical (e.g., skills) and social (e.g., culture) changes and improvements may be achieved. These changes and improvements can then lead to improved efficiency and productivity. Efficiency and productivity can then improve cost-management. Better cost-management and control can then lead to cost-reduction, cost-minimisation, cost-avoidance, cost-containment, and cost-saving. All these are outputs, immediate and intermediate outcomes that become mechanisms through which monetary ROI is achieved. Before then, the outputs present as non-monetary returns-on-investments either as enabled abilities (e.g., cost-management, cost-reduction, cost-minimisation, cost-avoidance, cost-containment), outputs or intermediate outcomes (e.g., improved behaviour, productivity, efficiency).

Non-monetary ROI can also be achieved through organisational development e.g., staff development and collaboration. Organisational development is the basis for safe healthcare systems and may lead to cost-saving, and hard cash ROI. Improvements in staff and process outcomes may improve culture, which may also improve patient and financial outcomes. Improvements in patient outcomes may lead to further benefits (e.g., incentives), and become an organisation’s legacy (culture, capacity and capabilities). This can help an organisation become more resilient and sustainable. QI culture and QI legacies are the basis from which future organisational development as well as patient and financial outcomes can be achieved.

Altogether, the QI outcomes contribute to higher goals such as organisational learning, transformation, financial stability, value-based healthcare, and high reliability [101, 102, 105, 116]. Although intended goals and short-term outcomes may be achieved earlier, long-term sustainable impacts depend on successful implementation, embedding a QI safety culture, and developing legacies that support future improvement efforts. Whatever the end-outcome, lessons may be learnt, research, innovation and development may ensue, capacities and capabilities may improve. As Banke-Thomas et al. [68] stated, “ The application of (S)ROI … could be used to inform policy and practice such that the most cost-beneficial interventions are implemented to solve existing (public health) challenges” (p.10).

Figure 5 illustrates the updated QI-ROI conceptual framework in a phased format. This figure represents the current conceptualisation of QI-ROI based on our analysis of the healthcare QI evaluation literature. The processes described here are more complex but have been simplified for clarity. The figure contains the ROI-like concepts from our first study (e.g., efficiency, productivity, effectiveness, cost saving). These concepts are seen here as building blocks of financial ROI. However, some of these also form part of improvements in other organisational performance and developmental goals. Such improvements can be seen as non-monetary ROI which includes improved abilities, development, and overall improved outputs and outcomes. Together, these are the building blocks of the QI-ROI concept as indicated by the literature.

## Discussion

The aim of this part of the review was to further develop a framework for understanding the benefits that reflect the concept of ROI from large-scale healthcare QI programmes (the QI-ROI). We achieved this by reviewing different QI literatures on the goals and or benefits from QI programmes. The goals embody aspirations or QI-ROI as imagined, whilst the reported outcomes and benefits represent QI-ROI as experienced. Together, these form a concept of QI-ROI. We considered negative outcomes to be part of this conceptualisation as they may highlight perceptions of the absence of the QI-ROI. We grounded our theoretical assumptions on organisational needs, duties, and obligations as defined by organisations themselves as well as various internal and external stakeholders.

Our assumption was that at a minimum, a QI programme that delivers on any organisational needs and obligations, delivers a return-on-investment for healthcare organisations. The reviewed literature revealed numerous QI goals and outcomes. These included aspects of an organisation’s performance and development, as well as external and unintended QI outcomes. Through the Complexity Theory lens, we noted the different connections of these outcomes. This deepened our understanding of QI-ROI as a collection of interlinked QI benefits that occur incrementally throughout a programme’s lifecycle. These benefits include systems, processual, and structural improvements. Central to these, are sustainable improved patient outcomes.

Although QI effectiveness was not the focus of this review, it is related to QI-ROI. In-fact some view ROI as an overall measure of QI effectiveness [22]. Since the induction of QI into healthcare, a sizeable body of literature have questioned QI’s value and effectiveness [136–142]. Several factors have been found to determine QI’s success. These include aspects of organisations’ structures, systems, behaviours, cultures, and leadership [143, 144]. The collection of benefits referred to in this review as QI-ROI largely contribute towards these QI effectiveness determinants [145–147]. Thus, improvement in these aspects must be of value for organisations. Further, achieving QI’s pre-defined goals (effectiveness) is not the end, but part of the journey towards QI-ROI. This is important to note as depending on the QI resources required, costs may increase, rendering QI value inversely related to its cost [21, 148, 149].

The insights into the building blocks of good quality healthcare are not new and inter-disciplinary health services research attest to this [150–153]. Wider health and social science as well as organisational literature have repeatedly pointed to the importance of improving staff capacities and capabilities, as well as experience [154]. A systematic review by Hall et al. [155], found that poor staff wellbeing and burnout are frequently associated with poor patient outcomes. Latino [156] argued that the intellectual capital of human beings is one of the greatest benefits not captured through financial outcomes. Implementation and Improvement Sciences have highlighted the importance of understanding contexts, interventions, and human behaviour and their influence on QI programme success and sustainability [39, 40].

Effective leadership was a consistent patient safety pre-requisite in the Francis Mid-Staffordshire review [157]. The Francis review also highlighted negative cultures and failure to learn as contributing factors to poor quality care. Negative QI outcomes and failed attempts must be avoided, but they are part of learning safety cultures [158]. Patient engagement has also been found to be crucial in leaning and safety cultures [159]. A safety culture: one that prioritises safe care, is thus deemed foundational to efforts to improve quality and safety [158, 160–164].

There are of-course other ways to improve healthcare, and organisations do invest in various programmes that specifically target some of the themes within our QI-ROI conceptual framework, for example leadership programmes [165]. Determining whether QI or other types of investments and programmes led to any specific improvement is known to be challenging [166, 167]. As a result, claims of causality are not possible. Through Complexity Theory, QI-ROI can be viewed in terms of contribution or correlation to organisational outcomes rather than direct attribution [11, 37, 166]. Understanding of QI contribution to organisational outcomes may be achieved through methods such as contribution analysis and the action effect method [166, 167]. These methods can help detect the type and level of QI contribution.

QI’s key contributions to healthcare improvement are evident in the reviewed literature, and external bodies such as the UK Care Quality Commission (CQC) attest to this. In 2018, 80% of Trusts rated “Outstanding” by the CQC had organisational improvement programmes [101]. As a result, QI was identified in the UK National Health Service (NHS) Long-term Plan as an approach for improving every aspect of how the NHS operates [168]. Further, organisations that have mature improvement cultures claim to have benefited in several of the QI-ROI conceptual framework’s dimensions [169–171]. Mature organisations indicate that, in addition to organisational development and performance, environmental and social impacts [172], reputation, [173], and resilience [174], are crucial organisational outcomes. QI programmes are now also used to engage with modern healthcare agendas like value-based healthcare and environmental sustainability [175, 176]. In achieving such goals, QI programmes can be cost-effective without saving actual costs [177].

However, QI-ROI is not a one-time event. ROI may be created or lost at different stages of a programme [25]. In a complex healthcare programme, QI-ROI is iterative and dynamic with many determinants, some of them outside the control of QI implementers alone [13, 39, 167]. Additionally, QI may affect various levels of stakeholders from frontline, to societies, to policymakers differently. [13, 39, 167]. These levels interact with and influence each other [11, 39]. As such, it is important to note the co-dependencies of QI outcomes when planning and evaluating QI. As Donabedian [178] stated; structures, processes and outcomes are mutually dependent. This means that it is important to take small wins with big wins through observing the QI-ROI chain [179]. Therefore, not only is the traditional ROI approach unreliable as a forecasting tool, as an evaluation tool, it is a distal and an incomplete marker of QI value.

Finally, large-scale programmes took many forms, some internal and some involving external collaborators. Collaborations have been recommended as a way to improve patient safety and experience, and save costs [180, 181]. However, unless formally integrated, organisations run internal budgets, their performance assessed individually, and with own governance structures [14, 182–184]. Notably, collaboratives appear to be geared towards health system-wide benefits and indirectly address organisational-level impacts [138]. Therefore, collaboratives may bring unique challenges as well as benefits. This may mean that different organisations at different developmental levels deduce different outcomes from the same QI programmes [102, 146]. Research developments here will be valuable to improve understanding of QI-ROI, for example how and why collaboratives work (or not) [51, 185]. Nonetheless, this review reveals largely shared QI goals and outcomes regardless of the type of large-scale programme.

### Strengths and limitations

A strength of our review is that our theoretical assumptions were grounded on organisational needs, duties, and obligations as defined by organisations and external stakeholders. This step preceded the first study where we analysed different returns-on-investments concepts in healthcare QI. The current study sought to strengthen the first study’s QI-ROI conceptual framework by connecting the QI-ROI concept with categories of QI benefits as seen by healthcare QI stakeholders. Additionally, our review lens through complexity theory gave us a glimpse of the processes though which these QI-ROI building blocks independently or in concert may influence ROI. As such, our framework provides clues to its practical application.

A limitation of this review is that it was broad, encompassing various disciplines in various countries, reporting on different types of programmes. The review was meant to be an exploration of the QI field’s view of QI returns-on-investment. Researchers may wish to explore these in specific contexts, for example by studying particular “building blocks” of QI-ROI in a specific context or programme. Additionally, some of the literature is quite dated, however newer literature do suggest continuance of some trends and issues in QI-ROI and QI business case matters. Lastly, subjectivity in the synthesis and analysis cannot be ruled out as it is inherent in qualitative analyses [63].

### Implications for research and practice

Economic evaluation of large-scale programmes are a new phenomenon, and research is needed to help identify the most suitable evaluation methods. This need is compounded by the fact that large-scale QI programmes come in many forms. It is important to assess QI’s contribution to organisational performance and development through suitable and innovative research methods such as realist reviews rather than seek a definitive causal link which may be imperceptible in complex large QI programmes. A study of collaboratives alone or in comparison to internal organisation-wide QI programmes may help explore the best ways to approach large-scale QI programmes to maximise ROI. In addition, a thorough study of the relationships of the QI-ROI determinants as well as QI benefits may help to understand why and how QI benefits influence one another. Lastly, guidance on how to weigh different QI benefits, and how to develop a standardisable yet flexible QI-ROI tools will be crucial for future research and practical application.

## Conclusion

ROI in healthcare is a highly debated topic. This review is but one contribution to this ongoing debate. Our review suggests that in healthcare, ROI must reflect value-based healthcare principles, with value defined as patient and organisational benefits. We hope that by defining the ROI concept in this manner, links between wider large-scale QI benefits and organisational strategic intents will be highlighted. In doing this, leaders may be able to frame QI value, benefits and thus ROI in a useful way. This broader view is crucial if healthcare organisations and health systems are to continue investing in essential healthcare quality improvements. ROI is not a one-time event and may be created or lost at different stages of a programme. Further, many factors determine whether it can be deduced, many of them outside the control of QI implementers. Such factors must be taken into consideration in valuing healthcare QI.

## Supplementary Information

**Additional file 1: Supplementary Table 1.** Example search strategy. **Supplementary Table 2.** Data extraction tool. **Supplementary Table 3.** Included studies. **Supplementary Table 4.** Summary of Quality assessment. **Links** Current study PRISMA Checklist. Search strategies. Data extraction tool. Excluded studies.

## Data Availability

The datasets used and/or analysed during the current study are available from the corresponding author on reasonable request. Some data has been included in this published article as its supplementary information files.

## Abbreviations

QI: Quality Improvement
ROI: Return on Investment
SROI: Social Return on Investment
QI-ROI: Return on Investment from healthcare nhquality improvement
CEA: Cost Effectiveness Analysis
CUA: Cost Utility Analysis
CBA: Cost Benefit Analysis
