# Supplementary material for: Identifying and understanding benefits associated with return-on-investment from large-scale healthcare Quality Improvement programmes: an integrative systematic literature review
Source: BMC Health Serv Res. 2022 Aug 24;22:1083. doi: 10.1186/s12913-022-08171-3 (PMC9404657; doi:10.1186/s12913-022-08171-3)
Supplement: Supplementary file 1 — Additional file 1: Supplementary Table 1. Example search strategy. Supplementary Table 2. Data extraction tool. Supplementary Table 3. Included studies. Supplementary Table 4. Summary of Quality assessment. Links Current study PRISMA Checklist. Search strategies. Data extraction tool. Excluded studies. [file 12913_2022_8171_MOESM1_ESM.docx]

**Supplementary table 1: Example search strategy**

**Web of Science**: ti.ab.kw

| **Concept 1: Context** | **Concept 2: QI Methods** | **Concept 3: QI Outcomes** | **Limits** | **Database** |
| --- | --- | --- | --- | --- |
| Health*    All levels | "Quality improvement" OR QI OR “statistical process control” OR Lean OR “Six sigma” OR “Lean* Six-sigma” OR Audit NEAR/1 feedback OR “Model for improvement” OR “Root cause analysis” OR “Process mapping” OR Define NEAR/1 Measure NEAR/1 Analy?e NEAR/1 Improve NEAR/1 Control OR DMAIC OR “Plan do study act” OR PDSA OR PDCA OR “Driver diagram” OR “Theory of change” OR “Logic model” OR “statistical quality control” OR SQC | Return NEAR/1 investment OR “Rate of return” OR  Payback OR “Business case” OR Benefit* NEAR/1 cost OR Risk* NEAR/1 benefit* OR Cost* NEAR/1 benefit* OR Cost* NEAR/1 consequence* OR “Cost reduction” OR “Cost containment” OR “Cost control” OR “Cost avoidance” OR Cost* NEAR/1 saving* OR cost* NEAR1 outcome* OR Value NEAR/1 investment OR Value NEAR/1 care OR “Value for money” OR Value NEAR/1 improvement OR Improvement NEAR/1 outcome* OR Resource* NEAR/5 outcome* OR Resource* NEAR/5 benefit* | None | Web of Science: (All databases)   - MEDLINE - SciELO Citation Index - Russian Science Citation Index - Web of Science Core Collection - KCI-Korean Journal Database |

Link: <https://apps.webofknowledge.com/UA_CombineSearches_input.do?product=UA&SID=F5tdD2WX3IIMXwfjJfW&search_mode=CombineSearches>

**Supplemental table 2 Data extraction tool**

| Author & Country | Country | Setting | Type of article | Type of programme | ROI/Outcomes/Impact discussed |
| --- | --- | --- | --- | --- | --- |
| Bailit and Dyer 2004 | US | - | Conceptual | General QI  Business case | Business case framework: organized around three broad areas: direct financial considerations, strategic considerations, and internal organizational considerations:  Patient, staff, organization, external stakeholders, financial outcomes, direct and indirect, image, reputation, competitiveness, legal, patient enrollment, compliance with performance and quality requirements, status (license or influence), accreditation, oversight, incentives (carrot and stick), recruitment and retention, alignment with mission/ethical |
| Banke-Thomas et al. 2015 | UK | Public Health | Review  ROI studies | ROI in Public Health | Socio-economic, environmental, stakeholder engagement, patient outcomes |
| Beers et al. 2017 | US | Paediatric Mental Health Primary Care  19 practices; 8 academic health centers, 6 private practices, 4 federally qualified health centers (FQHC), 1 outpatient specialty clinic.‍ | QI Evaluation  Report | Improve screening practices in primary care.‍  PDSA | Community resources, Health care financing, Support for children and families, Clinical information system redesign, Decision support for clinicians, sustainability, cost-effectiveness,  ? negative consequences |
| Benning et al. 2011 | UK | 22 NHS hospitals; 4 interventions, 18 control  general wards, critical care, perioperative care, and management of medicines | Mixed methods study  Before & After  Quantitative (surveys)  Qualitative (interviews, document analysis) | Safer Patients Initiative  Independent evaluation  PDSA | Patient outcomes, clinical processes,  staff engagement, QI leadership development/effectiveness, systematization of QI, sustainability of QI, organizational learning, compliance, penetration, sustainability, risk management, safety culture, raising awareness about capacity issues, negative consequences related to staff engagement and implementation |
| Bevan et al. 2011 | UK | - | Conceptual  Large-scale change | General large-scale QI | Leadership, patient outcome, organizational development (capacity and capability), QI spread and sustainability, productivity, efficient use of staff, cost saving |
| Bielaszka-DuVernay 2011 | US | Hospital; Two acute care units | Report | Collaborative: Redesigning Acute Care Processes in Wisconsin  Lean | Financial outcomes, patient satisfaction, process outcomes, defining clinician roles, efficiency (resources and staff), clarifying roles, patient engagement, improved communication-staff and patients, new ways of working, structures and processes, professional development, pride, productivity, effectiveness, spread, risk management, negative consequence-penalised for moving pts too quickly |
| Bosse et al. 2015 | Tanzania | Three hospitals, 1 int, 2 control  surgical departments  hernia and varicocele, appendicitis, intestinal obstruction, and septic wounds | Quantitative  Before and after | Improving pre and post op care using checklist  PDSA, Donabedian SPO | Structural and process outcomes, clinical outcomes, contribution to curricula, sustainability, |
| Botros and Dunn 2019 | UK | Hospital; Surgical department; urology, upper and lower gastrointestinal surgery, vascular surgery, and orthopaedics | Action research  Implementation | Medicine’s reconciliation  PDSA | Clinical processes, patient safety, QI fidelity, spread and sustainability, staff and patient communication and relationships, supporting staff education, efficiency (staff time, variation, waste), innovation, compliance with standards, raising awareness/education, process outcomes, governance-performance management drs, improved multidisciplinary working, effectiveness, |
| Bridges 2006 | US | - | Conceptual  Patient perspective | Lean vs HTA | Efficiency and safety of a system, innovation, universal access. ethical and social consideration stakeholder engagement, organizational culture, risk management, |
| Bryan et al. 2019 | UK | - | Conceptual  (Discussion and guide) | General large-scale QI | Patient safety, Efficiency, clinical processes, culture and climate, leadership and governance, skills and workforce, infrastructure, and resources, innovation, sustainability, integration and collaboration, strategic/mission alignment, capability and capacity, |
| Brink et al. 2017 | South Africa | 34 hospitals obstetric and gynaecological, orthopaedic, cardiovascular, thoracic, and other vascular surgery, neurosurgery, and gastrointestinal surgery | Quantitative  Pre &Post | Reducing Surgical Site Infections (SSIs)  Audit & Feedback | Process outcomes, clinical outcomes, new ways of working, role clarification, highlighting additional areas of improvement, compliance, collaboration and teamworking |
| Care Quality Commission  2018 | UK | - | Conceptual  (Report) | General QI | Patient safety, staff development, leadership, culture development, systems strategy |
| Chow-Chua and Goh 2002 | Singapore | - | Conceptual | General QI  ROI | **QI and performance evaluation framework**  Patient, staff, organizational benefits. |
| Ciarniene et al. 2019 | Lithuania | - | Conceptual  Literature review, Qualitative study, Document analysis | General QI | QI patient value conceptual framework  Patient and organizational benefits. |
| Collins and Fenney 2019 | UK | - | Conceptual  (Discussion) | Collaboratives  Review | Patient safety, organizational culture, leadership development, staff development, efficiency |
| Comtois et al. 2013 | Canada | Hospital | Report  Economic evaluation  Observation and document review; Report | Hospital-wide QI review 2006-2011  Kaizen/Adapted Six-Sigma | Financial outcomes, patient outcomes, new ways of working, highlighting related areas of improvement, efficient time use, staff satisfaction, cumulative cost savings, leadership development, empowerment, align with health reform, building foundation for bigger complex, innovation |
| Crawley-Stout et al. 2016 | US | Public health | Quantitative; Before and after ROI calculation | QI experiential learning (QI 101) programme  Lean | Financial benefits; saving time, process improvement, clinical effectiveness leading to reduced A&E attendance, and improved smoker diagnosis, staff development, informed patients, innovation, external/community benefits. |
| Crema and Verbano 2017 | Italy | - | Systematic Review | - | Patient safety, process management, clinical effectiveness, cost-efficiency, ethical issues (fairness, appropriateness, rights, solidarity), priority-setting |
| De Miranda et al. 2020 | Brazil | Hospitals (state, municipal and national) | Quantitative  Before & after | Healthcare Associated Infections (HCAIs) programme  PDSA | Leadership, Regulations and standards, Organizational capacity (educational and training, Information, Population participation |
| de la Perrelle | Australia  Multinational | - | Review (of) QI collabs.  Economic review QI Collaboratives | QI collaboratives | Financial outcomes, clinical outcomes, process outcomes, staff engagement, development of guidelines, patient and public engagement, patient retention/increase, cost and time savings to carers/patients, cost-effectiveness, improvements in other conditions, spreading costs and benefits, off-setting other benefits, |
| DelliFraine et al. 2010 | US  Multinational | - | Review | Lean Six-Sigma Review | Clinical outcomes, process outcomes, financial outcomes (cost effectiveness) |
| Fischer et al. 2020 | US | - | Conceptual  Explains ROI and related | General QI  Business case | Patient safety and experience, financial benefits, processes, external stakeholders e.g., society, aligning values and priorities, team engagement, data, Maximize current people and systems, recruitment, retention, reputation, incentives, accreditation, regulation, capacity, throughput, productivity, future costs, performance management |
| Fortney et al. 2012 | US | Outpatient clinics  Different specialties | Feasibility study  Non-Randomised  Implementation | Collaborative: telemedicine based CCM program to improve depression care. PDSA | Clinical outcomes, process of care, implementation outcomes |
| Furukawa et al. 2016 | Brazil | Hospital  Pharmacy and a medical-surgical clinic | Report | Environmentally sustainable medication process  Lean | Benefit to the institution, environment, and health. Raising awareness, training and education, IT improvement, new ways of working, role clarification, |
| Gandjour & Lauterbach 2002 | Germany | - | Conceptual | General QI | Financial benefits, social benefits, clinical outcomes |
| Goodridge et al. 2008 | Canada | Province-wide | Quantitative  Survey | Retrospective survey  Lean | Nurse engagement, ownership, normalization, cultural shift, leadership development, training and education, professional and career development, |
| Hatcher 2002 | US | Hospital | Report | Needle-stick injury  PDSA | Clinical outcome, financial savings, staff well-being |
| Heitmiller et al. 2010 | US | Department of Anesthesiology and  Critical Care Medicine | Quantitative  Lean Sis-Sigma | Reducing blood product wastage  Lean Six Sigma | Raising awareness, education and training, multidisciplinary teamworking, processes and structural changes, cost-saving, innovation, staff engagement, patient safety, change in practice, role clarification and accountability, innovative research method. |
| Honda et al. 2018 | Brazil | - | Review | Lean Six-Sigma review | Patient safety and experience, increased patients, clinical outcomes, staff costs and turnover, efficiency, process improvement |
| Hunter et al. 2015 | UK | 14 sites; primary care trusts, provider trusts including mental health, community, acute care, and ambulance services. | Mixed methods  Mixed Qual  ITS | Northeast Transformation System (NETS) | Clinical outcomes, clinical processes, staff engagement, staff development, |
| Kanamari et al. 2015 | Senegal | Health Centre: 9 departments | Qualitative | 5S Pilot staff perceptions study  Lean | Work environment, attitude and behavior of staff, attitude and behavior of patients, and quality of services (efficiency, patient-centeredness, and safety). |
| Lavoie-Tremblay et al. 2017 | Canada | Multi-hospital  8 units | Report | Transforming Care at the Bedside Program  PDSA | Patient experience, clinical outcomes, team effectiveness |
| Leatherman et al. 2003 | US | 4 case studies business case discussion | Conceptual: 4 case studies. Lit. review  Interviews  Expert opinion  Document analysis | General QI  Business case | Financial benefits, enhanced market position; reduced regulation and oversight; improved reputation; improved patient retention and decreased reenrollment, marketing, and acquisition costs; improved recruitment and retention of essential staff; and improved health outcomes, pride, market share, |
| Masso et al. 2010 | Australia | New South Wales healthcare  10 hospitals | Qualitative  Senior managers interviews | Clinical Services Redesign Program (CSRP).  Lean Six-Sigma | Cultural change, leadership development, performance management, sustainability, influence, legitimacy amongst peers, alignment with priorities, |
| McGrath et al. 2017 | UK | 4 Hospitals: ICU & ENT | Report | Global Tracheostomy Collaborative  PDSA | New ways of working-ne teams/collaborations, clarifying organizational goals, staff engagement, patient engagement, clinical outcomes, education, training, awareness, leadership development, use of data infrastructure, reputation, litigation, financial outcomes, service status, influence, financial incentives, innovation. |
| McVane et al. 2019 | Multi-country  Sweden Malawi, Bulgaria, Indonesia  2 more | - | Conceptual  Review of 6 articles from the International Journal of Health Governance | Lean healthcare governance  Lean | Clinical outcomes, patient safety and experience, staff engagement and empowerment, leadership development, organizational culture, stakeholder engagement, efficiency, infrastructure development |
| McLees et al. 2015 | US | Public health  Different specialties | Conceptual  Expert opinion, Literature review, review of quality award data | General QI | QI evaluation framework  Efficiency and effectiveness (includes organizational outcomes e.g., development and processes), staff satisfaction |
| Mery et al. 2015, 2017 | Canada | Multinational review | Review  System-wide QI  (Collaboratives?) | Capacity building QI  ROI | **Framework for evaluation:**  Organisation capacity and capability, High performance, self-sustaining, effective resource allocation, programme spread and sustainability, staff capacity and capability, patient outcomes, financial outcomes |
| Morganti et al. 2012 | US | 40 healthcare organisations | Conceptual  Document analysis  Interviews | Perfecting Patient Care (PPC)  Toyota Production System | Spread and sustainability, patient outcomes, staff development, culture |
| Moraros et al. 2016 | Canada | - | Review | Lean effectiveness | Patient experience, clinical outcomes, staff outcomes, financial outcomes |
| Morrow et al. 2012 | UK | Hospitals; 96 organisations  5 case studies | Mixed methods; Interviews and survey | The Productive Ward: Releasing Time to Care  Lean | Teamwork and collaboration, staff engagement, career development, skill development, financial benefits, patient safety and experience, innovation, leadership development, clinical structures and processes, better use of data, sustainability, |
| Neri et al. 2008 | US | Virtual Health  Multi-hospital | Report | Blood product utilization  Six-sigma | Patient safety, financial outcomes, clinical processes |
| Niemeijer et al. 2015 | Netherlands | Hospital | Review  Report | 5 Year impact of Lean Six Sigma | Increase number of admissions Improve ward/department capacity.  Improve productivity of personnel Reduce unnecessary use of diagnostic tests.  Patient satisfaction Improve safety.  Reduce costs by reducing inventory.  Increase revenue Improve utilization of equipment by use of ICT Improve process of purchase and maintenance -Improve utilization of outpatient clinic. Increased influence, improved purchasing procedures and bargaining power, Efficient use of staff-redirecting, |
| O’Sullivan et al. 2020 | UK | Hospital | Report | General QI | Training and development, staff motivation strategic alignment, service user engagement, clinical outcomes, patient experience, leadership development, organizational development, sustainability |
| Pearson et al. 2017 | UK | Regional health and social care | Quantitative  ITS | Hospital at home  PDSA | Stability, Creating foundation for future QI, collaboration and teamworking, ownership, motivation, process of care, sustainability, improved relationships, patient safety. |
| Perencevich et al. 2007 | US | - | Conceptual  Discussion | Hospital Acquired infections business case. | Business case framework  Externalities, reputation, incentives, benefits to untargeted patients, patient safety, legal costs, |
| Power et al. 2016 | UK | Hospitals:  10 regions  133 hospitals | Report  Mixed methods  Action research | Harm Free Care  Four harms; venous thromboembolism (VTE), pressure ulcers, urinary tract infection in patients with urinary catheters and falls. PDSA | Alignment with national goals, system and process, collaboration, networking, engagement, sustainability, initiative fatigue, establishment of a measurement system, patient safety, development of the NHS safety thermometer, strategy refinement, financial incentives |
| Robert et al. 2020 | UK | 8 Hospitals | Qualitative-mixed method | 10-year review of the Productive ward collaborative programme  PDSA & Lean | Patient outcomes, staff outcomes, implementation outcomes, influence, structures and processes, culture, |
| Rogers et al. 2008 | Australia | - | Conceptual  ‘Proof of concept’ | The Stronger Families and Communities Strategy | Cost-benefit methodology  Financial /non-financial, short-term/long-term, positive/negative |
| Roney et al. 2016 | US | Hospital: 11 units | Report | Implementation of a MEWS‐Sepsis screening tool  PDSA | Clinical outcomes, implementation outcomes, staff engagement |
| Schouten et al. | Netherlands | Diabetes management:  Hospital outpatients and family medicine | Controlled before & after | Collaborative  PDSA | Health care costs, Health outcomes (QALYs), clinical outcomes, process outcomes, social outcomes (carers and families) |
| Shah and Course 2018 | UK | Hospital  Case studies | Conceptual | General QI  Business case/ROI | The ELFT framework for evaluating return on investment from quality improvement: evenue, Cost reduction Cost avoidance  Productivity and efficiency Staff experience  Patient, carer, and family experience outcomes, high reliability, team efficiency and productivity, staff turnover, sustainability, spread, acquisition of new business, influence through reputation, using data more rigorously, market share, support other organisations, registration status, |
| Sibthorpe et al. 2018 | Australia | Public health | Review | Primary care Aboriginal community  PDSA | Patient clinical outcomes, process outcomes, systems outcomes |
| Sermersheim et al. 2020 | US | Hospital: Adult ICU, Paediatric ICU, Emergency department | Report | Improving Patient Throughput with an Electronic Nursing Handoff Process  FOCUS-PDSA | Process outcomes, organizational influence, spread and sustainability, standardized pathways, innovation, meeting external obligations, leveraging existing systems, teamworking, culture, education and training |
| Staines et al. 2015 | Sweden | Jönköping County Council health departments | Qualitative interviews and document analysis | 20 Year review of QI | Patient satisfaction, clinical outcomes, financial outcomes, influence, sustainability, quality award, structures and processes, leadership development, recruitment, organizational resilience, culture, innovation, education, and research, pride, incentives, patient engagement, |
| Stephens et al. 2018 | UK | 93 Hospitals: surgery, anaesthesia, and critical care | Report | The Enhanced Peri-Operative Care for High-risk patients (EPOCH) trial  PDSA | 93 Hospitals: surgery, anaesthesia, and critical care, Enhanced resources, QI skill delivery, patient outcomes, process outcomes, fidelity |
| Strauss et al. 2019 | Canada | Hospital | Quantitative before and after | Choosing wisely  Reductions in unnecessary aspartate aminotransferase and blood urea nitrogen tests; Audit & Feedback | Systems and Process outcomes, sustainability, cost saving, training, and education |
| Swensen et al. 2013 | US | 22 hospitals  Case studies | Conceptual  Expert opinions over experiential track record of 22 hospitals | General QI  Business case | QI evaluation framework: the needs of patients, & reputation, & esprit de corps, and & financial return sufficient to maintain state-of-the-art medical practices. Alignment with patient interests, moral obligation, patient safety, reliability, staff engagement, legal, society, sustainability, agreement on shared goals, new ways of working, finding other related systems/process defects, collaboration, benefits to employers e.g return to work, leadership development, redefining and clarifying roles, productivity, multi-hospital collaboration, improving quality curricula, spread/diffusion, cost-effectiveness, create business case for better data management, creating patient trust to lower legal costs, job security, trust staff and leadership, quality and academic influence, future cost avoidance, new essential staff roles, sustain and accumulate, |
| The Health Foundation 2011 | UK | NHS Hospitals; general ward care, critical care, peri-operative care, and medicines management. | Conceptual  (Technical report) | Safer Patients Initiative? Breakthrough Collaborative  PDSA/Lean/SPC | Clinical outcomes, clinical processes, organizational culture, and climate, spread, safety skills and awareness, design for patient safety, high reliability, networking and collaboration, staff engagement, empowerment, leadership engagement, teamworking. |
| Thursky et al. 2018 | Australia | Hospital | Mixed methods  Exploratory sequential design | Sepsis management  Process Mapping | Clinical outcomes, structural and process outcomes, spread and sustainability, financial outcomes. |
| Van den Heuwel et al. 2006 | Netherlands | Hospital | Conceptual | General QI  Six Sigma | Cost reduction, quality improvement, and patient safety. Increased market share, process outcomes, cross departmental effects |
| Wells et al. 207 | UK | -, | Economic eval. Review  20 yrs. of QIC | Breakthrough Collaboratives, Keystone Collaboratives | Effectiveness, cost-effectiveness, sustainability, share data, innovations, learn faster, more effective in implementing and spreading improvement ideas, improve processes of care and if sustained, improve patient outcomes, and reduce healthcare costs. powerful way to scale up and spread innovations and create long-term learning networks, compliance with performance criteria, team engagement, culture change, aligns with values and drives intrinsic motivation, data management, data |
| White et al. 2014 | UK | Hospital | Review | Productive Ward-Releasing Time to Care. Lean | Patient safety, staff well-being, financial savings, sustainability. |
| Williams et al. 2020 | UK | Hospital | Quantitative  naturalistic stepped- wedge trial | Productive Ward-Releasing Time to Care  PDSA | Patient outcomes, friends and family outcomes, nursing teams outcomes-culture and resilience, doctor-patient communication |
| Wood et al. 2019 | UK | Hospital and community  secondary care, community services, care homes and the ambulance service. | Report  Quantitative (survey) | Quality Improvement Collaborative (QIC)  Reducing pressure ulcers  PDSA | Clinical outcomes, process outcomes, staff engagement, staff empowerment, patient and family engagement, financial savings |
| Worral et al. 2008 | UK | 4 Mental Health organisations | Realist Evaluation  Report | Mental Health Improvement Partnerships programme  PRINCE 2 | Social inclusion. Patient outcomes, staff engagement, training, and education, learning and innovation, collaboration, organizational capacity, financial outcomes, culture development. |
| Yamamoto et al. 2010 | US | Hospital: emergency department (ED), medical-surgical unit, intensive care unit (ICU)/progressive ICU (PICU), and burn unit, pharmacy manufacturers | Report  Before & after | Improving Insulin Distribution and Administration Safety  Lean Six Sigma | Clinical outcomes, patient safety, service user experience/satisfaction, efficiency of time use, cost-saving, process outcomes, |

**Supplementary table 3: Included studies**

| Author & Country | Country | Setting | Type of article | Type of focus | Programme Type | Outcomes category |
| --- | --- | --- | --- | --- | --- | --- |
| 1.Bailit and Dyer 2004 | US | ---------- | Conceptual | Economic | QI Business Case Guideline development | 1(a, b, & c),2, |
| 2. Banke-Thomas et al. 2015 | UK | Public Health | Systematic Review: Economic; SROI | Economic | Social Return on Investment (SROI) in Public Health | 1a, 2, 3a+ |
| 3. Beers et al. 2017 | US | Paediatric primary care: 19 practices; 8 health centers | Quantitative (Longitudinal) | Non-Economic | Improve screening practices in primary care: Plan-Do-Study-Act (PDSA) | 1(a & b), 2, 3a-, 3b |
| 4. Benning et al. 2011 | UK | 22 NHS hospitals; 4 interventions, 18 control | Mixed methods: (Surveys, interviews, document analysis) | Non-economic | Safer Patients Initiative: PDSA | 1(a & c), 2, 3b |
| 5. Bevan et al. 2011 | UK | ---------- | Conceptual | Non-economic | QI Guide to improvement/transformation | 1 (a & c), 3b |
| 6. Botros and Dunn 2019 | UK | Hospital; 5 specialties surgical | Quantitative (longitudinal) | Non-economic | Medicine’s reconciliation: PDSA | 1 (a, b, &c), 2, 3a+. 3a-, 3b |
| 7. Bielaszka-DuVernay 2011 | US | Hospital; Two acute care units | Brief Report | Non-economic | Collaborative: Redesigning Acute Care Processes in Wisconsin: Lean | 1 (a, b, &c), 3a+, 3a- |
| 8. Bosse et al. 2015 | Tanzania | Three hospital, surgical depts | Quantitative (Pre & Post) | Non-economic | Improving pre and post op care using checklist: PDSA | 1a & c ,3b |
| 9. Bridges 2006 | US | ---------- | Conceptual | Non-economic | General QI Discussion | 1 (a, b, & c), 3a |
| 10. Brink et al. 2017 | South Africa | 34 hospitals; 8+ specialties | Quantitative (Pre &Post) | Non-economic | Reducing Surgical Site Infections (SSIs) Audit & Feedback | 1 (a & c), 3a |
| 11. Chow-Chua and Goh 2002 | Singapore | ------------- | Conceptual | Non-economic | QI Evaluation Framework Development | 1 (a, b, & c), 2 |
| 12. Ciarniene et al. 2019 | Lithuania | ------------- | Conceptual (Literature review, Qualitative study, Document analysis) | Non-economic | QI evaluation Conceptual framework development | 1 (a, b, & c), 2 |
| 13. Collins and Fenney 2019 | UK | ------------ | Conceptual | Non-economic | Collaboratives Reflective Review and Discussion | 1 (a, b, & c), 2, 3a+, 3a- |
| 14. Comtois et al. 2013 | Canada | Hospital | Economic evaluation: Economic Impact (Observation and document review) | Economic | Hospital-wide QI impact review 2006-2011: Kaizen | 1 (a, b, & c), 3 a+ |
| 15. Care Quality Commission 2018 | UK | ----------- | Conceptual | Non-economic | CQC Grading Progress Report | 1 (a & c), 2, 3a+, 3a-, 3b |
| 16. Crawley-Stout et al. 2016 | US | Public health | Economic evaluation: ROI | Economic | QI Experiential Learning (QI 101) programme: Lean | 1 (a, b, & c), 2, 3a+ |
| 17. Crema and Verbano 2017 | Italy | ----------- | Systematic Review | Non-economic | Lean Management to support Choosing Wisely | 1 (a, b, & c), 2, 3a+, 3b |
| 18. De Miranda et al. 2020 | Brazil | Hospitals (state, municipal and national) | Quantitative (Pre &Post) | Non-economic | Healthcare Associated Infections (HCAIs) programme  PDSA | 1 (a, b, & c), 2, 3a+, 3a-3b |
| 19. de la Perrelle | Australia | ------------ | Systematic Review: Economic | Economic | General QI | 1 (a, b, & c), 2, 3b |
| 20. DelliFraine et al. 2010 | US  Multinational | ------------ | Literature Review | Non-economic | Lean Six-Sigma Review | 1 (a, b, & c), 3a- |
| 21. Fischer and Duncan 2020 | US | ----------- | Conceptual | Economic | General QI Discussion | 1 (a, b, & c), 2 |
| 22. Fortney et al. 2012 | US | Outpatient clinics: Different specialties | Mixed Methods (routine data, surveys, interviews) | Non-economic | Collaborative: telemedicine based program to improve depression care: PDSA | 1 (a, & c), 3b |
| 23. Furukawa et al. 2016 | Brazil | Hospital: Pharmacy and a medical-surgical clinic | Quantitative (Pre & Post) | Non-economic | Environmentally sustainable medication process: Lean | 1 (a, & c), 2, 3a+ |
| 24. Gandjour & Lauterbach  2002 | Germany | ---------- | Conceptual | Economic | General QI Discussion | 1 (a & b), 2 |
| 25. Goodridge et al. 2008 | Canada | Province-wide | Quantitative (Survey) | Non-economic | The implementation processes associated with Lean | 1 (a, & c), 2, 3a- |
| 26. Hatcher 2002 | US | Hospital | Brief Report | Non-economic | Needle-stick injury: PDSA | 1 (a, b, & c) |
| 27. Heitmiller et al. 2010 | US | Anesthesiology & Critical Care Medicine | Quantitative (Pre & Post) | Non-economic | Reducing blood product wastage: Lean Six Sigma | 1 (a, b, & c), 3a+ |
| 28. Honda et al. 2018 | Brazil | --------- | Systematic Review | Non-economic | Lean Six-Sigma | 1 (a, b, & c) |
| 29. Hunter et al. 2015 | UK | 14 sites; primary care trusts, and ambulance services. | Mixed methods (Qualitative, document analysis, Interrupted time series (ITS)) | Non-economic | North-East Transformation System (NETS) | 1 (a, b, & c), 3a+, 3a-, 3b |
| 30. Jones et al. 2019 | UK | ----------- | Conceptual | Non-economic | Large-scale QI Discussion and guideline | 1 (a, b, & c), 2 |
| 31. Kanamari et al. 2015 | Senegal | Health Centre: 9 departments | Qualitative | Non-economic | 5S Pilot: Lean | 1 (a, b, & c) |
| 32. Lavoie-Tremblay et al.  2017 | Canada | Multi-hospital: 8 units | Quantitative (Pre & Post, time-series) | Non-economic | Transforming Care at the Bedside Program: PDSA | 1 (a, & c) |
| 33. Leatherman et al. 2003 | US | --------- | Conceptual (Literature review, Interviews; Expert opinion, Document analysis) | Economic | General QI business case knowledge synthesis | 1 (a, b, & c), 2 |
| 34. Masso et al. 2010 | Australia | New South Wales healthcare: 10 hospitals | Qualitative (Interviews) | Non-economic | Clinical Services Redesign Program (CSRP).  Lean Six-Sigma | 1 (a, b, & c), 2, 3a+, 3a-3b |
| 35. McGrath et al. 2017 | UK | 4 Hospitals: ICU & ENT | Quantitative (longitudinal) | Non-economic | Global Tracheostomy Collaborative: PDSA | 1 (a, b, & c), 2, 3a+ 3a- |
| 36. MacVane et al. 2019 | Multi-country | --------- | Conceptual (Review of 6 articles from the Int. Journal of Health Governance) | Non-economic | Lean healthcare governance: Lessons from Lean application | 1 (a, b, & c), 3a+, 3b |
| 37. McLees et al. 2015 | US | Public health: Different specialties | Conceptual (Expert opinion, Literature review, award data) | Non-economic | QI outcomes framework development | 1 (a, b, & c) |
| 38. Mery et al. 2015, 2017 | Canada | --------- | Systematic Review: SROI | Economic | QI capacity building ROI framework development | 1 (a, b, & c), 2, 3 a+, 3b |
| 39. Morganti et al. 2012 | US | 30 healthcare organisations | Conceptual (Survey, routine data, interview | Non-economic | Comparing self-reported and externally rated QI success. | 1 (a, & c), 2, 3b |
| 40. Moraros et al. 2016 | Canada | --------- | Systematic Review | Non-economic | Lean effectiveness: Lessons from Lean application | 1 (a, b, & c) |
| 41. Morrow et al. 2012 | UK | Hospitals: 96 organisations, 5 case studies | Mixed methods (Interviews & survey) | Non-economic | The Productive Ward: Releasing Time to Care: Lean | 1 (a, b, & c), 2, 3 a+ 3a- 3 b |
| 42. Moody et al. 2015 | Netherlands US |  | Conceptual | Economic | SROI Lessons from the Netherlands and US | 1 (a, b, & c), 2 |
| 43. Neri et al. 2008 | US | Virtual Health: Multi-hospital | Quantitative (longitudinal) | Non-economic | Blood product utilization: Six-sigma | 1 (a, b, & c), 2, 3a+, 3b |
| 44. Niemeijer et al. 2015 | Netherlands | Hospital | Conceptual | Non-economic | 5 Year impact of Lean Six Sigma: Service Review Report | 1 (a, b, & c), 2, 3 a+ |
| 45. O’Sullivan et al. 2020 | UK | Hospital | Conceptual | Non-economic | General QI: Service Review Report | 1 (a, b, & c), 3a+, 3-, 3b |
| 46. Pearson et al. 2017 | UK | Regional health & social care | Quantitative (Interrupted Time Series) | Non-economic | Hospital at home: PDSA | 1 (a & c), 2, 3 a+ |
| 47. Perencevich et al. 2007 | US | --------- | Conceptual | Economic | Hospital Acquired infections business case guideline | 1 (a, b, & c), 2 |
| 48. Power et al. 2016 | UK | 133 Hospitals; 10 regions | Mixed methods (interview, observations, survey, documents) | Non-economic | Harm Free Care: Four harms; PDSA | 1 (a, b, & c), 2, 3a+ 3a- 3b |
| 49. Robert et al. 2020 | UK | 8 Hospitals | Mixed-methods interviews, survey, questionnaires, observations | Non-economic | 10-year review of the Productive ward collaborative programme: PDSA & Lean | 1 (a & c), 2, 3b |
| 50. Rogers et al. 2009 | Australia | ---------- | Conceptual | Economic | Methodology for a qualitative cost–benefit evaluation | 1 (a, b, & c) |
| 51. Roney et al. 2016 | US | Hospital: 11 units | Brief Report | Non-economic | Implementation of a MEWS‐Sepsis screening tool: PDSA | 1 (a & c), 3b |
| 52. Schouten et al. 2010 | Netherlands | Hospital outpatients and family medicine | Economic evaluation: Cost effectiveness | Economic | Diabetes management: Collaborative: PDSA | 1 (a, b, & c), 2 |
| 53. Shah and Course 2018 | UK | Hospital | Conceptual | Economic | QI ROI Framework development | 1 (a, b, &c), 2 |
| 54. Sibthorpe et al. 2018 | Australia | Public health | Systematic Review |  | Primary care Aboriginal community: PDSA | 1 (a, b, & c), 2, 3b |
| 55. Sermersheim et al. 2020 | US | Hospital: AICU, PICU, ED | Brief Report | Non-economic | Improving Patient Throughput with an Electronic Nursing Handoff Process: FOCUS-PDSA | 1 (a & c), 2, 3a+ 3a- 3b |
| 56. Staines et al. 2015 | Sweden | Jönköping County Council health departments | Qualitative (multimethod) | Non-economic | 20 Year review of QI | 1 (a, b, &c), 2, 3a+ 3a- 3b |
| 57. Stephens et al. 2018 | UK | 93 Hospitals: surgery, anaesthesia, critical care | Mixed-Methods (routine data, ethnography, survey | Non-economic | The Enhanced Peri-Operative Care for High-risk patients (EPOCH) trial: PDSA | 1 (a & c), 2, 3a- 3b |
| 58. Strauss et al. 2019 | Canada | Hospital | Quantitative (Pre & Post) | Non-economic | Choosing wisely: Reductions in unnecessary aspartate aminotransferase, blood urea nitrogen tests: Audit & Feed | 1 (b, & c), 3b |
| 59. Swensen et al. 2013 | US | ---------- | Conceptual | Economic | QI ROI Framework development | 1 (a, b, & c), 2 |
| 60. The Health Foundation 2011 | UK | NHS Hospitals; 4+ specialties | Conceptual | Non-economic | Lessons from Safer Patients Initiative: PDSA/Lean/SPC | 1 (a & c), 2, 3a+ & 3b |
| 61. Thursky et al. 2018 | Australia | Hospital | Mixed methods (routine data, focus groups) | Non-economic | Sepsis management: Process Mapping | 1 (a, b, & c), 3a+ |
| 62. Van den Heuwel et al. 2006 | Netherlands | Hospital | Conceptual | Non-economic | General QI Guideline: Six Sigma | 1 (a, b, & c), 2, 3 a+ |
| 63. Wells et al. 2017 | UK | ---------- | Systematic Review: | Non-economic | Breakthrough Collaboratives, Keystone Collaboratives | 1 (a, b, & c), 2, 3a+ & 3b |
| 64. White et al. 2014 | UK | Hospital | Literature Review | Non-economic | Productive Ward-Releasing Time to Care: Lean | 1 (a, b, & c), 3a+ 3a- 3b |
| 65. Williams et al. 2020 | UK | Hospital | Quantitative (naturalistic stepped- wedge) | Non-economic | Productive Ward-Releasing Time to Care: PDSA | 1 (a, & c), 2, 3a+ 3a- 3b |
| 66. Wood et al. 2019 | UK | Hospital and community, and ambulance service. | Quantitative (survey) | Non-economic | Quality Improvement Collaborative (QIC)  Reducing pressure ulcers: PDSA | 1 (a, b, & c), 2, 3b |
| 67. Worral et al. 2008 | UK | 4 Mental Health Organisations | Conceptual | Non-economic | Mental Health Improvement Partnerships programme: Comprehensive programme report | 1 (a, b, & c), 2, 3a+ 3a- 3b |
| 68. Yamamoto et al. 2010 | US | Hospital: 5 specialties and pharmacy manufacturers | Quantitative (Pre & Post) | Non-economic | Improving Insulin Distribution and Administration Safety  Lean Six Sigma | 1 (a, b, & c) |

**Supplementary table 4: Summary of Quality assessment**

SQUIRE 39 Items; 12 categories. STaRI 27 Items; 7 categories. CHEERS 24 Items; 6 categories. CASP 10 items. MMAT 5 items each

A mixture of quality assessment and reporting tools. No formal way to reconcile rating of studies; thus high, medium, poor based or reported and assessed items.

| HIGH 80-100% n=15 (39%) | | | MODERATE 50-79% n=16 (43%) | | POOR <50% n=7 (18%) |
| --- | --- | --- | --- | --- | --- |
| Banke-Thomas (CASP)  Benning (MMAT)  Crema (CASP)  de la Perrelle (CASP)  Goodridge (MMAT)  Fortney (STaRI)  Wells (CASP)  Moraros et al. 2016 (CASP) | Hunter (MMAT)  Masso (MMAT-Qualitative)  Robert (MMAT)  Schouten (CHEERS)  Wood (SQUIRE)  Yamamoto (SQUIRE)  Staines (MMAT-Qualitative) | Botros (STARI)  Heitmeiler (SQUIRE)  Honda (CASP)  McGrath (SQUIRE)  Power (SQUIRE)  Sibthorpe (CASP)  Pearson (SQUIRE)  Bosse (SQUIRE) | | Strauss (MMAT-Quantitative)  Thursky (STaRI),  Williams (MMAT)  Morrow ?(MMAT)  Weiner (MMAT)?  Kanamori et al. 2015 (MMAT-Qualitative)  Beers (SQUIRE)  Brink (SQUIRE) | Neri (SQUIRE)  Comtois (CHEERS)  Crawley-Stout (CHEERS),  de Miranda (STaRI)  Lavoie-Tremblay (MMAT)  Mery (CASP)  Furukawa et al. 2016 (MMAT) |

File link: <https://onedrive.live.com/?id=D1DBD5A64FD65ECB%212748&cid=D1DBD5A64FD65ECB>

**Links**

Current study PRISMA Checklist

<https://onedrive.live.com/?id=d1dbd5a64fd65ecb!0*L0xpdmVGb2xkZXJzL0RvY3VtZW50cy9ST0kgUmV2aWV3IENoZWNrbGlzdC5kb2N4>

Search strategies

<https://onedrive.live.com/?cid=D1DBD5A64FD65ECB&id=D1DBD5A64FD65ECB%21629&parId=D1DBD5A64FD65ECB%21105&action=defaultclick>

Excluded studies

<https://onedrive.live.com/edit.aspx?resid=D1DBD5A64FD65ECB!10910&ithi=nt=file%2cdocx&authkey=!AGlxIOZgoPXWA_I>
